# Analysis of Metagenomic Data Containing High Biodiversity Levels

**DOI:** 10.1371/journal.pone.0058118

**Published:** 2013-03-07

**Authors:** José R. Valverde, Rafael P. Mellado

**Affiliations:** 1 Scientific Computing Service, Centro Nacional de Biotecnología (CSIC), c/Darwin, 3, Madrid, Spain; 2 Department of Microbial Biotechnology, Centro Nacional de Biotecnología (CSIC), c/Darwin, 3, Madrid, Spain; Wageningen University, The Netherlands

## Abstract

In this paper we have addressed the problem of analysing Next Generation Sequencing samples with an expected large biodiversity content. We analysed several well-known 16S rRNA datasets from experimental samples, including both large and short sequences, in numbers of tens of thousands, in addition to carefully crafted synthetic datasets containing more than 7000 OTUs. From this data analysis several patterns were identified and used to develop new guidelines for experimentation in conditions of high biodiversity. We analysed the suitability of different clustering packages for these type of situations, the problem of even sampling, the relative effectiveness of Chao1 and ACE estimators as well as their effect on sampling size for a variety of population distributions. As regards practical analysis procedures, we advocated an approach that retains as much high-quality experimental data as possible. By carefully applying selection rules combining the taxonomic assignment with clustering strategies, we derived a set of recommendations for ultra-sequencing data analysis at high biodiversity levels.

## Introduction

The analysis of microbiological biodiversity has advanced significantly with the inclusion of Next Generation Sequencing (NGS) technologies [1]. While early biodiversity studies relied on relatively small sample sizes to estimate biodiversity in the population [2], [3], [4], modern sequencing technology allows data retrieval from possibly thousands or even millions of microorganisms, hence rendering more reliable and comprehensive studies [5]. This capability can be further exploited by combining several samples in a single experiment using tag-encoded amplicon pyrosequencing [6], which permits subsequent binning of data to samples.

The quality of the different NGS technologies and their suitability for environmental studies is a recurrent subject in specialised and comparative reviews [7], [8], [9], [10], [11]. Most studies use Roche 454 technology to obtain the sequences, due to its capacity to produce a large number of longer reads: up to a million reads, with lengths ranging from 300 to 500 base pairs, and a well-characterized, non-uniform mean error rate of 1%. Recently, interest in using Illumina technologies has increased as well as in the corresponding read lengths [12], [13], [14], [15]. Various approaches have been developed to deal with NGS-induced errors [16], including the removal of reads with ambiguous base calls [8], de-noising using various approaches, pre-clustering, different methods for chimera detection, and the removal of singleton sequences (ones that do not cluster with other sequences, resulting in OTUs with a unique member sequence) [17] or sequences below a given abundance threshold [18], [14].

Typical experiments consist of sequencing 16S rDNA using specially targeted primers, something particularly well suited for this approach as it contains several hypervariable regions that can accommodate a large diversity [19]. Most studies are based on sequencing only a fragment containing one or more hypervariable regions, usually the V6, V3–V5, V4–V6 or V9 regions. Traditionally, bacterial biodiversity has been measured by estimating the number of culturable species present in the population, however, it is now possible to collect genetic information from unculturable organisms, which frequently cannot be ascribed to characterised species. Deciding whether these novel sequences should be assigned to a new species raises the controversial issue of correspondence between taxonomy and genetic variability. Operational Taxonomic Units (OTUs) at 3%, 5% and 10% dissimilarity are commonly used to estimate richness as regards species, genus and phylum [20].

Recommendations for data analysis from NGS experiments have recently been made [16] as a Standard Operation Procedure (SOP) that uses a very demanding approach, discarding any potentially questionable sequence and keeping only those whose quality can be precisely verified. This large reduction in reads available for study is justified when dealing with low diversity environments, offering the great advantage of ensuring that only the highest quality data is used to obtain the estimation. A different approach consists of removing all reads with less than three copies when the number of reads remaining is large enough to guarantee saturation [14], however, in this type of analyses, richness estimators such as Chao1 cannot be used and so they must resort to rarefaction analysis to verify richness saturation. A more conservative approach using an abundance threshold has been proposed to manage highly complex communities: a large number of high quality reads differ by only 1 nucleotide from the expected sequence, with variants occurring at every position, suggesting they are sequencing errors [18], [13]. This approach allows inclusion in the analysis of sequences that are not fully conserved. Therefore, the approach chosen to filter and analyse the sequences exerts a major impact on the results as each method has its own strengths and weaknesses, and hence the choice of an adequate approach is not an easy matter. The most restrictive approaches are certainly an excellent option for low-diversity samples, however, there are circumstances where richness estimation can potentially require up to several hundred thousand reads [21]. A less restrictive solution would facilitate the analysis of combined samples in high-diversity situations, however, little work has been conducted to determine recommendations for adequate sample sizes considering major factors such as expected biodiversity, shape of the population's relative species abundance distribution and expected minimal OTU composition. Indeed, current studies usually include minimal analyses to understand relative OTU distribution in the underlying populations, often reporting Shannon-Weaver's biodiversity index or using long-standing controversial methods, such as pie charts [22].

The selection of the best tools to analyse different NGS datasets is the subject of active research and many comparative analyses usually consider datasets of relatively limited size and variable levels of diversity [23], [24], [25]. A performance comparison of the commonly used tools on data sets with a very high diversity will provide useful guidance for the selection of the appropriate tool. Our own research focuses on the analysis of samples from agricultural soils treated with different herbicides, or supporting different types of crops. In principle, we expect these environments will harbour more complex bacterial communities with a potentially high degree of diversity. Careful thought should be given to ensure that less represented, yet possibly relevant, species in the target environments are not being neglected. Considering all these points, we set out to study in more detail various issues that are specific to the analysis of NGS samples from complex environments, aiming to obtain useful strategies to retrieve the maximum amount of information on the underlying biodiversity with minimal cost in reads. We started by comparing the relative efficiency of the various tools used to calculate the OTUs; we then sought to better understand the effect of the sampling and population structure by analysing the behaviour of the observed and predicted OTUs on carefully designed synthetic populations and subsequently we considered the additional noisiness of real-world situations using experimental datasets from a variety of sources, leading us to propose a cost-effective work flow for data analysis. This may have a major impact on the development of agricultural strategies, management and policies.

## Materials and Methods

### Datasets

To better understand the expected behaviour of the current estimators and methods, we produced two synthetic communities, where the total sequences in each one had been derived from a single, well-known reference sequence (*E. coli* full-length 16S rRNA (1542 nt) and its V3–V5 region (592 nt)) by using EMBOSS msbar [26] to add random point mutations (transitions, transversions, insertions and deletions), until a distance of approximately 3.5% was achieved (16S-20K and V3V5-20K data sets). All the sequences were compared using NCBI-blast [27] to identify the number of groups present at 3% dissimilarity.

A second set of communities was generated from reference sequences available from VAMPS [28] (retrieved on 2012-03-05) using the complete V3, V3–V5, V4–V6, V6, V6a, V9 regions and full-length SSU. The reference databases were first clustered using Otupipe [29] to identify representative sequences from separate OTUs at a 3% distance; these were further cleaned by comparison using BLAT [30] to obtain a list of sequences for each reference database with a distance of at least 3% among them. We selected 10000 sequences from these in each case (7000 in the case of V6) to generate communities following reference log-normal distributions produced with R [31], and parametrized using various values for μ and σ (the mean and standard deviation of the logarithm). New individuals were added to each OTU using EMBOSS msbar to mutate each seed sequence to a distance of 1.25% the number of times specified by the chosen distribution (refV3, refV3V5, refV4V6, refV6, refV6a, refV9 and refSSU datasets).

Rarefaction analysis of these datasets will simulate sampling from a natural community where most OTUs have only one individual. To simulate a situation where each OTU has more than one individual in the community, we built additional communities with each OTU containing at least two or three individuals, by duplicating and triplicating the datasets (x2 and x3 datasets). Full details on the procedures used in building the synthetic communities are provided in File S1.

Finally, we have considered several published experimental datasets from different environments: data obtained from agricultural soil samples [32], from different grasslands [33], from Priest Pot lake [34] and faecal samples [35]. These datasets are considered to represent situations with an anticipated high or medium diversity, and with sampling sizes that are characteristic of the current 454 multiplexed experiments. All datasets are available from the authors at the web site http://www.free-bit.org/public/metagenomics/.

### Data Analysis

When dealing with experimental datasets we relied on UCHIME as a cost-effective approach to remove chimeras [16]. We applied chimera removal early in our pipeline using Otupipe, both checking with the Gold reference database and using frequency counts [29]. The reported chimeras were then removed from the dataset prior to further analysis.

To obtain comparable results in each case, we applied the same cleaning procedure described in the original publication to each experimental dataset, except for the faecal samples, where no undetermined bases (N) were allowed.

To identify eukaryotic, putative contaminants and questionable sequences, we included a taxonomical analysis step. Seeking maximum accuracy, NCBI-blast was used to search reads against a reference database. Initially, we used the RDP [36] and Silva databases [37], however, as comparisons against Silva ran significantly faster and offered more sensitive assignments, subsequent analyses relied only on comparisons with Silva. The search output was then processed with MEGAN [38] to assign reads to the different taxa.

We employed the taxonomical classification computed by MEGAN to separate the reads into three groups: sequences clearly belonging to the group of interest (in our case bacterial sequences), sequences clearly identified as contaminants (in our case only eukaryotic sequences, as no archaea reads were identified in any of the experimental datasets analysed) and unclassified reads (all others). Unclassified reads were then clustered separately to identify singletons and these were discarded as questionable sequences. Unclassified reads remaining after the removal of singletons were then pooled back with the group of interest and the definitive analysis was performed on the sum of both (in our case, unclassified reads minus singletons plus bacteria).

The initial OTU analysis was carried out using the RDP pipeline, ESPRIT [39], MOTHUR [40], and Otupipe [29]. More detailed subsequent analyses were carried out using Otupipe with a minimum cluster size of 1. The output of Otupipe was processed to extract the relevant information (File S1), such as chimerical sequences, sequences clustered in singleton or doubleton groups, relative richness and abundance analysis, and to calculate the corrected value for Chao1 and ACE. Lastly, rarefaction curves were built using Otupipe and the QIIME pipeline tools [41]. The process was automated employing easy-to-use, self-explanatory, command-line programs available on GitHub (http://github.com/jrvalverde/MGtools) and listed in SeqAnswers (http://seqanswers.com/wiki/CNB_MetaGenomics_tools). These are provided in File S1.

## Results

### Analysis of Synthetic Datasets

The preliminary analysis was carried out using the “simplified” communities (obtained by generating random mutations from a single original sequence), 16S, V3V5 and their duplicated and triplicated versions (x2 and x3). To obtain an accurate estimate of the diversity present we used NCBI-blast to compare each sequence with all the others. The comparisons revealed that these datasets contained 8647 and 8399 artificial OTUs at 3% dissimilarity, respectively. As a first preliminary analysis step, we compared the relative efficiency of the different tools (ESPRIT, MOTHUR, RDP pipeline and Otupipe) to calculate the number of OTUs at 3% dissimilarity of all the simplified ommunities. For comparison purposes all runs were performed using a single processor. In all cases, the analyses underwent an earlier comparison step to reduce data complexity that consistently identified one representative sequence for each synthetic OTU, reducing further calculation: ESPRIT and MOTHUR correctly identified the number of seed sequences in all the 16S and V3V5 datasets. Subsequent execution times were therefore independent of the number of sequence copies in a community. However, there were major differences among the different methods: subsequent analysis in ESPRIT required the computation of a combinatorial number (37,380,981 for 16S*-derived comminities) of pairwise Needleman-Wunsch comparisons to build the distance matrix needed to perform the clustering, thus explaining its long run time (over two weeks); MOTHUR, instead, built a multiple sequence alignment which, although less expensive, still required a long time (over a week), while the RDP pipeline and Otupipe, used different approaches that coped with large biodiversity datasets very efficiently (minutes to hours), becoming more suitable for these type of problems. For the sake of convenience, Otupipe was selected as the tool of choice in all the subsequent analyses.

**Figure 1:**
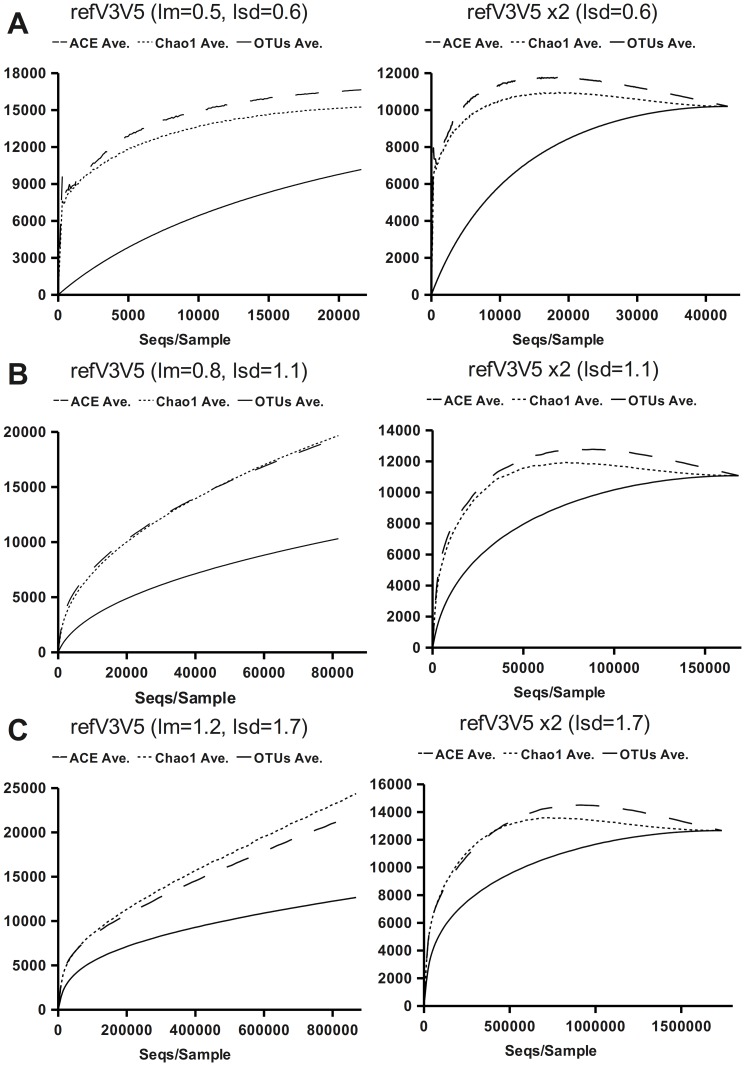
Analysis of full-length 16S rRNA synthetic data. The data for various populations derived from VAMPS reference V3V5 database constructed using various values of μ and σ is shown in graphs A, B and C, respectively. The left pane shows rarefaction curves for the base population, and the right pane shows the curves for the derived duplicated population. Solid lines represent observed OTUs at 3% dissimilarity, dotted lines represent the number of OTUs predicted by corrected Chao1 and dashed lines represent the number of OTUS predicted by ACE. Increasing OTU membership results in greater values of μ.

The results obtained from the synthetic communities highlight some relevant trends in usual data analysis situations (Figure 1 and Figure S1) that are confirmed irrespective of whether the analysis was carried out on simplistic or more variable VAMPS-based communities.

First, the response observed was the same in all cases, irrespective of the 16S regions used, and dependent only on the generated community parameters.

Second, the total number of OTUs was only observed when all, or almost all, individuals in a community had been analysed. This was also the case when every OTU had more than one member sequence: although one might initially expect to have sampled all OTUs at least once previously, the randomness implicit in the sampling process renders this unlikely.

Third, regarding richness estimators, both the ACE and corrected Chao1consistently tended to overestimate richness and gave similar estimates in the base communities, Chao 1 being more accurate when σ<1 and ACE when σ>1, with Chao1 giving better results when the communities contained two or more individuals on each genetic lineage. Both ACE and the corrected Chao1 increased rapidly at the start, with ACE producing an early peak (better appreciated in Figure 1A), reaching a maximum value above the actual community richness and subsequently decreasing continuously until the actual richness was matched.

Fourth, the effect on the community parameters of increasing OTU membership by duplicating or triplicating the datasets is reflected as an increase in μ with minimal changes in σ.

Finally, when the OTUs were allowed to have only one individual present in the community (base communities), both produced a final value that grossly overestimated the actual richness (as expected, given the predictive weight they assign to singletons), even when all the individuals in the community were covered. However, when we used the duplicated and triplicated communities (Figure 1, Figure S1), OTU sampling evened out and the estimators reached an acceptable estimate earlier.

The point where estimators begin to reach a plateau and converge to the actual richness might be used to estimate a minimum sampling size. Our analyses showed that this plateau depends on expected biodiversity, community relative richness distribution and minimum number of individuals present in the community from any given OTU.

### Analysis of experimental data sets

Experimental data are needed to gain a deeper understanding of data analysis in situations where a large diversity is anticipated. We selected four different kinds of datasets: in the data sheet from the Priest Pot lake [34] the diversity was estimated using over ten thousand short-length sequences of the V5 16S rRNA region. The second kind of datasets consisted of soil samples from different types of grasslands where diversity was estimated using a higher number of sequences (around thirty thousand) of the larger V2–V3 16S rRNA region [33]: the datasets FMG1, FUG1, UPG1 and UPG3 were selected as representing datasets with different ratios of diversity to sample size. The third kind corresponded to soil samples collected in 2011 from maize cultivars where the small size V6 16S rRNA region was sequenced [32]: we selected the largest sample corresponding to non-Bt-maize at the final sampling time (SF4, 36314 sequences) and, since basically no differences were found in the structure of the corresponding rhizobacterial communities, pooled it with data from non-Bt-maize at the first sampling time (SF2+SF4, 49646 sequences) and from Bt-maize at the final sampling time (SF3+SF4, 63516 sequences) to obtain larger datasets. Finally, a fourth kind comprised faecal samples from children, which were taken to study gut microbiota using the V5–V6 hypervariable 16S rRNA region [35]: we chose to analyse the largest samples: ERR011058 (10BF), ERR011062 (12BF) and ERR011080 (5EU) (21811, 25724 and 22714 sequences, respectively), with the first two (−58 and −62) also displaying higher diversity (about 2000 OTUs).

**Figure 2:**
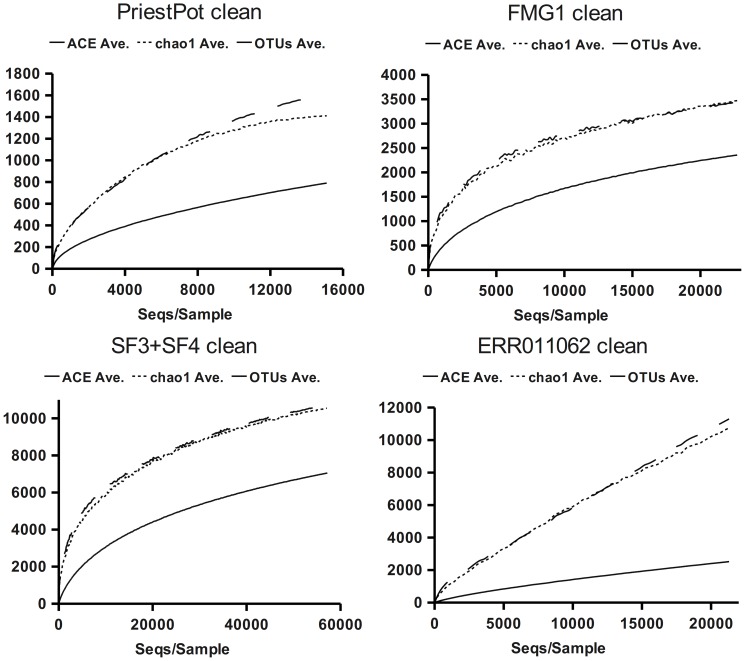
Rarefaction curves from experimental data sets. Evolution of ACE, Chao1 and OTUs figures with the sample size. The continuous line represents the OTUs distribution at 3% dissimilarity, the dotted and dashed lines represent the evolution of Chao1 and ACE respectively, with sample size. The selected datasets are Priest Pot lake, grassland FMG1 pooled SF3+SF4 maize rhizosphere and ERR011062 faecal sample to measure diet influence on gut microbiota; all were analysed after cleaning and chimera removal.

While the synthetic datasets provide an insight into an ideal situation, the actual field data carries a significant payload due to the random influences at various steps in the analysis, from initial sampling, to purification, amplification and sequencing, all of which may introduce an error in the estimation. To reduce these errors, all the experimental datasets were filtered, as described by the original authors, and to satisfy the minimal length constraints required by the RDP pipeline, except for the faecal samples, where no undetermined bases (N) were allowed. Additionally, as the presence of chimeras is a non-negligible source of error, we applied a chimera removal step immediately after, by means of analysis with Otupipe and the removal of all reported chimeras. The remaining sequences were analysed to calculate the rarefaction curves for observed and predicted OTUs at 3% dissimilarity. For the sake of simplicity, we herein report one dataset of each kind. Figure 2 shows the graph of observed and predicted OTUs. To better appreciate the final slope of each curve, the number of reads and CHAO1 predictions for the last ten points are provided in Table 1 and Table 2. The results for all the datasets are reported as Figure S2 and Table S1.

**Table 1:** Average values of Chao1 for selected datasets.

Priest Pot clean		FMG1 clean		SF3+SF4 clean	
Chao1	N. reads	Chao1	N. reads	Chao1	N. reads
1395.93	14200	3404	21800	10451.83	56300
1402.31	14300	3437.71	21900	10449.89	56400
1407.13	14400	3448.59	22000	10451.13	56500
1404.39	14500	3435.2	22100	10472.97	56600
1407.18	14600	3449.63	22200	10472.27	56700
1402.42	14700	3444.21	22300	10469.55	56800
1412.22	14800	3463.74	22400	10480.05	56900
1406.92	14900	3468.51	22500	10475.89	57000
1410.13	15000	3465.08	22600	10478.49	57100
1411.76	15100	3472.3	22700	10486.24	57200

The average of the last ten observed values of Chao1 and their corresponding number of sampled sequences grouped for the selected datasets are listed to provide a more accurate picture of its evolution at the end of the rarefaction curve.

**Table 2:** Average values of Chao1 for selected datasets after removal of eukaryotic and questionable sequences.

Priest Pot -euk -uc1		FMG1 -euk-uc1		SF3+SF4 -euk -uc1	
Chao1	N. reads	Chao1	N. reads	Chao1	N. reads
934.2	13700	2659.53	21300	7473.65	54600
937.55	13800	2682.41	21400	7470.07	54700
927.57	13900	2678.11	21500	7482.71	54800
933.61	14000	2676.06	21600	7484.6	54900
928.62	14100	2675.58	21700	7470.57	55000
925.43	14200	2682.09	21800	7471.06	55100
925.07	14300	2682.38	21900	7482.87	55200
939.48	14400	2688.07	22000	7479.05	55300
930.85	14500	2687.22	22100	7481.15	55400
933.29	14600	2690.67	22200	7482.47	55500

The average of the last ten observed values of Chao1 and their corresponding number of sampled sequences grouped for the selected datasets after removal of eukaryotic and unclassified singleton sequences are listed to provide a more accurate picture of its evolution at the end of the rarefaction curve.

Despite quality filtering and chimera removal, we were still concerned about the hypothetical quality of the remaining sequences. Experimental data lack a reference standard with which to be compared, therefore, we resorted to analysing the taxonomic affiliations of the clean reads. We used an NCBI-blast search followed by MEGAN to classify the reads by NCBI taxonomy. A similar distribution was observed in all experimental cases (Table 3 and Table S2) where the vast majority of reads belonged to bacteria, as expected. We accepted these already filtered, high-quality sequences as “*bona-fide*” representing actual data from the samples, acknowledging that they could contain minor errors that are unlikely to affect the clustering analysis at 3% dissimilarity. No archaea sequences were identified in any dataset. Additionally, a minority of the sequences was assigned as belonging to eukaryotes and we assumed them to be contaminants that can be discarded considering their taxonomic affiliations and small number. Finally, about 10–20% of the sequences could not truly be assigned as bacteria or eukaryotes, according to MEGAN's criteria.

**Table 3:** Frequencies of taxonomic grouped sequences in each dataset.

Dataset	N	Eukaryota	Bacteria	Unclassified	U/N
synthetic 16S	20001	5	15486	4510	0.23
Priest Pot lake	15553	156	12591	2806	0.18
FMG1 grassland	23292	5	20855	2432	0.10
SF3+SF4 maize soil	59656	70	49353	10233	0.17

For each dataset the following data is provided: total number of sequences remaining after quality filtering and removal of chimeras and sequences that are too short (N), number of sequences identified as being of eukaryotic origin (Eukaryota), number of sequences identified as being of bacterial origin (Bacteria), number of sequences that could not truly be assigned (Unclassified) and proportion of unclassified sequences relative to the total number of sequences (U/N).

From the analysis of the synthetic datasets we learned of the importance of increasing the chances of genetic groups being sampled more than once. Unclassified sequences are a suitable target to retrieve additional information, yet at the same time care should be taken to eliminate any noisy sequence that could cause richness overestimation. Careful inspection of NCBI-blast alignments indeed showed that most of the unclassified sequences were very likely acceptable sequences and should be included in the study, although a small number of them were possible contaminants or sequences containing too many errors.

To avoid introducing biases in OTU computation, both contaminants and erroneous sequences should be discarded. We already knew from the taxonomic classification that contaminants are exceedingly rare and we expected these to be unlikely to cluster with other sequences. Regarding errors, a significant number of these need to be introduced for a sequence to be misclassified at 3% dissimilarity, to the order of more than 6–9 errors for 200–300 nucleotides-long sequences, and, since errors happen randomly, the likelihood of two sequences having the same error pattern is remote, implying that these erroneous sequences should appear as singletons. The other possible source of singletons in the unclassified reads group might be sequences belonging to hypothetical new species with a minute presence (one individual in tens of thousands) that are likely to exert a very small impact on the whole population and, in any case, although these remain as a possible source for the identification of new species, they are questionable and difficult to prove valid without additional experiments. For these reasons, we decided to carry out an additional clustering step on MEGAN's unclassified sequences, removing all singletons from them. The remaining sequences, which can be assigned in clusters with at least two individuals, were considered unlikely to be errors or contaminants, and were pooled with the already identified bacterial sequences to be used for further analysis.

**Figure 3:**
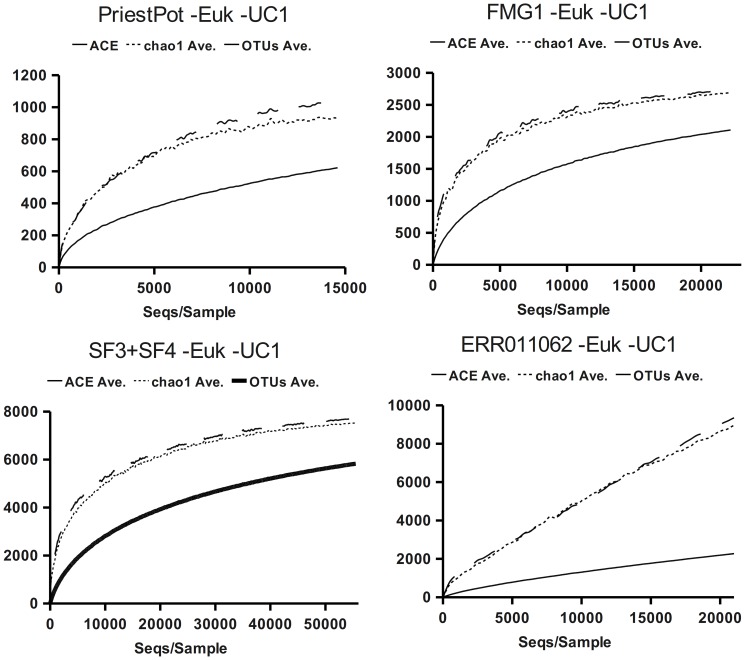
Rarefaction curves from experimental data sets (-Euk -UC1). Evolution of ACE, Chao1 and OTUs figures with the sample size. The continuous line represents the OTUs distribution at 3% dissimilarity, the dotted and dashed lines represent the evolution of Chao1 and ACE respectively, with sample size. The selected datasets are Priest Pot lake, grassland FMG1 pooled SF3+SF4 maize rhizosphere and ERR011062 faecal sample to measure diet influence on gut microbiota: results displayed were obtained after additional removal of eukaryotic sequences and unclassified sequences that clustered as singleton OTUs.

The resulting datasets were analysed using clustering at 3% dissimilarity to calculate the observed and estimated OTUs, and build rarefaction curves. The results for the selected datasets are summarised in Figure 3 and Table 1 and Table 2, and the total results are provided as supporting information, Figure S2 and Table S1. The values obtained after removing questionable sequences with the help of taxonomy are in all cases smaller that those previously obtained.

In the case of the Priest Pot data, where a moderate biodiversity value was expected and the starting data showed a σ of 1.4 (Table 4), the Chao1 estimator can be observed reaching the plateau with a relatively moderate sample size (the average slope of the last 10 rarefaction steps is −0.65). In the case of soil samples from grasslands, the expected biodiversity was higher and, although the rarefaction curve of the observed OTUs did not saturate biodiversity, the Chao1 estimate was already stabilised or close to stabilisation. When more sequences were considered and a higher biodiversity was expected, as in the case of pooled data from maize-culture soils, the effect of removing questionable sequences was more evident, both in the number of observed OTUs and in the behaviour of Chao1 estimates (Figure 3, Table 1 and Table 2). A special circumstance was observed in the case of faecal samples used to estimate gut microbiota: in this case from the rarefaction curves of the observed OTUs, Chao1 and ACE and from the distribution parameters it seems that we are still far from having adequate sampling to reach an accurate estimate of total richness. Chao1 generally produced lower estimates than ACE, even when σ>1.

**Table 4:** Log-normal parameter fitting of experimental data.

Dataset	μ	sd(μ)	σ	sd(σ)
PriestPot	0.97	0.05	1.42	0.04
FMG1	1.10	0.03	1.23	0.02
FUG1	0.93	0.02	1.14	0.02
UPG1	1.35	0.04	1.42	0.03
UPG3	0.95	0.02	1.19	0.02
SF4	0.98	0.02	1.15	0.01
SF2+SF4	0.96	0.01	1.12	0.01
SF3+SF4	1.01	0.01	1.16	0.01
ERR011058	0.38	0.02	0.86	0.01
ERR011062	0.40	0.02	0.90	0.01
ERR011080	0.66	0.04	1.28	0.03

The values estimated by fitting the richness distributions of each experimental data set to a log-normal distribution for the parameters μ and σ are provided in columns **μ** and **σ**. The estimated standard deviation for the estimation of each of these values is stated as **sd(μ)** and **sd(σ)**.

## Discussion

### Tools

ESPRIT is highly efficient in locating the relevant sequences using k-mer filtering, however, it requires very lengthy running times to build the pairwise alignments for high complexity data. A new version, ESPRIT-Tree, has been reported to provide significant improvements but is not yet available for production use, and has not been considered in this study. MOTHUR, which makes use of multiple sequence alignments and simple distance estimations, requires lower yet still lengthy running times. RDP and Otupipe, on the other hand, can work with the largest datasets in reasonable times with acceptable results. For this reason we consider that the latter tools are preferable when dealing with high complexity samples. The facility to locally run Otupipe presents a convenient advantage over the RDP web-based work flow when dealing with many datasets, albeit at the cost of requiring a suitably powerful computer. It is worth noting that QIIME bundles these (and many more) tools in a convenient way.

### Generation of synthetic communities

We have generated synthetic communities with a very large number of OTUs to stress test the analysis process. The preliminary analysis was conducted with simple synthetic communities generated under tightly controlled conditions: all individuals had “evolved” from a single reference sequence by accumulation of point mutations up to a 3.5% distance. We chose to build communities derived from the *E. coli* V3V5 region as representative of the current 454 read lengths, and from the full-length 16S as representative of an ideal situation, where all variability can be considered. These datasets were used to compare tool efficiency and draw initial estimates as to the complexity of the problem.

Experimental situations, however, may be better modelled by a more skewed log-normal distribution and display a wider variability. Furthermore, in real world experiments, regions other than V3V5 are also considered. To account for these factors, we generated communities derived from reference databases in VAMPS covering the most commonly used regions as well as the full-length SSU. It is well known that different organisms may not be resolved as separate OTUs when using a given region [42]. For this reason, we clustered all the sequences in each database to identify sequences representing separately identifiable OTUs. Since a large OTU may be split in the clustering process, it is still possible that some selected OTU representatives may be closer than 3% to each other. To avoid introducing a possible confusion factor to subsequent analyses, we then compared all representatives using BLAT (which is more efficient than Blast and just as reliable for distances below 3%) to obtain an initial set of sequences, each of which may act as a seed for a separate OTU. We next mutated selected seed sequences to generate the number of individuals required to match various log-normal distributions. The resulting synthetic communities represent an ideal situation where the whole region considered could be sequenced reliably from each sampled individual. Finally, when dealing with macroscopic organisms, it is possible for many species to be represented by only one individual in the community, however, when dealing with microorganisms, this requirement may be too restrictive, not only because the niche may accommodate a huge number of individuals, but also because their fast duplication pace renders the presence of genetically unique individuals more unlikely. These situations have been simulated by ensuring the presence of at least two or three copies of each synthetic sequence in the community.

Since we used the VAMPS databases, our synthetic communities do include specimens from all known phyla, as would be expected in real-world experiments. The main drawbacks of these communities are that in real experimental situations, mutation frequency varies across positions inside a given region, exhibiting base-pairing dependent coevolution, the whole region may not have been covered by the sequencing reads obtained, samples may have been enriched for specific phyla, and sequencing errors (which are usually cleaned up before the analysis process) may be encountered. In order to simulate these effects one would have to introduce additional uncertainty factors into the simulated communities, to the point where they would be undistinguishable from real experiments, and the reads would not be back-traceable any more. Hence, under these circumstances it makes more sense to relay subsequent analyses to actual experimental data.

### Data analysis

Initial analysis of simple communities helps to identify that sampling all existing genetically different specimens is very difficult, as indicated by the rarefaction curves of observed species in the x2 and x3 communities. This is still evident when VAMPS-derived populations are analysed, implying that to look for the saturation of observed OTUs alone may result in more expensive experimental designs, and to require sequences to be perfectly conserved more than once, or all OTUs to have a minimum number of members, may also require larger sample sizes, hence the use of richness estimators should generally be preferred.

We have compared the behaviour of corrected Chao1 and ACE in different situations. Both of them produce large overestimates when a population with many singletons is analysed. Less evident is their behaviour in what might possibly be a more common situation when dealing with bacterial biodiversity, i.e., that all OTUs have at least two or more individuals. Under these circumstances, employing random, incomplete sampling, we expect the predictors to produce more accurate results. Indeed, we can see that both predictors tend to reach a maximum estimate (higher when using ACE), and then slowly decrease to match the actual biodiversity value, once the whole population has been sampled. This behaviour depends on population complexity and the shape of the relative richness distribution. Two main routes may be derived from these results: firstly, according to our simulations, Chao1 behaves very well as a biodiversity estimator, and secondly, useful guidelines for estimating sampling size may be derived from studying the behaviour of a synthetic population with a biodiversity and log-normal distribution similar to those expected in the target population. However, we still have little knowledge with respect to actual population abundance distributions; our simulated populations may not cover the full range of distribution parameters and we assume that the whole region is sequenced, which is not always the case, especially as many species have very long hypervariable regions, and so, additional work is needed to derive useful rules for predicting appropriate sample sizes.

Our synthetic populations assume that reads used have been reliably sequenced, while in real-life experiments errors are introduced from a variety of sources. In this work to each experimental dataset we have applied the same filtering procedures originally reported by their authors to simplify the study and to obtain results that are more directly comparable with those already reported, the only exception being the datasets corresponding to human faecal samples, where we discarded reads with ambiguous bases (N).

Typically, the most stringent filtering processes will exclude a large amount of reads from an extensive sequencing effort, sometimes reducing their number to less than a tenth of the initial amount, and unique sequences to cluster to less than two orders of magnitude, yet still leaving enough reads to estimate biodiversity in environments with an expected low diversity.

Since we are concerned with the efficient use of runs when analysing samples with a relatively high diversity content, we took a different route to ensure keeping high-quality reads, while discarding the minimum amount of information possible. It is known that a large number of high-quality reads may include a minute number of random sequencing errors (to the order of 1%), which can generally be accommodated by the diversity allowed, according to the OTU definition.

We consider that chimera removal should be applied early in the analytical process, and certainly during the quality selection step, before definitive clustering takes place. UCHIME is reported to be a versatile and cost-effective tool [16], and given the efficiency of the Otupipe pipeline, we find it is worth running a preliminary clustering that will enable UCHIME to combine both frequency-based and Gold database reference comparison approaches for chimera detection. This initial step enables early detection and removal of chimeras from the dataset at a non-significant cost and may be used to gather useful information to estimate population distribution.

Combining taxonomic analysis with OTU clustering enables the inclusion of a larger number of additional reads in the analysis, potentially increasing its resolving power. We consider all reads that can be identified to be of bacterial origin as “*bona-fide*” sequences. This leaves about 10%–20% unclassifiable reads from which additional information can be retrieved. This group of unclassifiable reads can be considered as having acceptable sequences, unacceptable sequences containing errors, and questionable sequences that might (or might not) represent completely new species. The random distribution of sequencing errors can be used to discern acceptable sequences: if the number of errors is too high to permit their identification, it is extremely unlikely that any two erroneous sequences would display the same pattern, hence being reported as singletons after a clustering step of unclassified reads. Thus removal of singletons from the unclassifiable subset should mean the removal of all the unacceptable sequences. Arguably, this could eliminate some valid reads from new species, however, it is difficult to ascertain whether they are legitimate without additional experimental data, they represent less than one individual among tens of thousands in the population, and therefore we expect their functional contribution to be small, so that discarding them is most likely harmless.

Application of this protocol to experimental data produces OTU counts and estimates that are less affected by the presence of contaminants, chimeras and erroneous sequences, resulting in more rigorous estimates. When analysing faecal samples we detected a greater discrepancy between observed and estimated OTUs than previously reported [35], where a greater ambiguity (up to two N in the sequences) was allowed and different parameters, analysis workflow and software tools were used. It has been previously reported that these differences in the analytical procedure may lead to different results [24]. In any case, the significant difference between observed and estimated OTUs detected indicates a large presence of singletons that, when combined with the population parameters, points to the fact that we might be far from reaching saturation and measuring total alpha diversity.

The approach we have used to evaluate biological diversity is an inclusive one, and reflects the maximum number of reads that can reasonably be considered, without significantly distorting diversity estimation. As such, it allows for maximum efficiency in evaluating sequencing runs by reducing the amount of discarded sequences to the bare minimum, while only keeping reads that can be expected to be significant and correctly clustered at the same time.

## Supporting Information

Figure S1 Analysis of synthetic data. The data collected from all the synthetic datasets is summarized as rarefaction curves for observed OTUs at 3% dissimilarity, corrected Chao1 and ACE. Each row in the file contains the results for a given population and its duplicated and triplicated derivatives. The first two rows display the results obtained for the simplistic 16S and V3V5 populations, subsequent rows display the results for all VAMPS-derived populations arranged first by reference database (refV3, refV3V5, refV4V6, refV6A, refV6, refV9 and refSSU) and then by log-normal distribution parameters, from lower to higher σ. Yellow: observed OTUs average, red: Chao1 average, blue: ACE average, as a function of sample size.
(PDF)

Figure S2 Rarefaction curves from experimental data sets. Evolution of Chao1 and OTUs figures with the sample size. The dashed line represents the ACE average, the dotted line represents the Chao1 average and the continuous line the OTUs distribution at 3% dissimilarity. Left column (clean): results obtained after quality filtering and removal of chimeras and short sequences. Right column (-euk -UC1): results obtained after additional removal of eukaryotic sequences and unclassified sequences that clustered as singleton OTUs. Yellow: observed OTUs average, red: Chao1 average, blue: ACE average, at 3% dissimilarity and as a function of sample size.
(PDF)

File S1 Methods and tools for population generation and analysis. This archive contains the programs and scripts developed to perform the analyses described in the paper. All the scripts have been written for UNIX-like systems (such as Linux, MacOS X or Windows with Cygwin); they are self-documenting (when run with option ‘-h’) and are released under the EU-GPL license. Detailed descriptions of the protocols used and the rationale behind them are included as plain text files. Driver scripts to reproduce the generation and analyses of synthetic datasets are also provided. Third party tools and databases from external origin have not been included and should be obtained from their respective authors.
(ZIP)

Table S1 Average values of Chao1 for all datasets. The average and the last ten observed values of Chao1 and their corresponding number of sampled sequences grouped in each case are listed to provide a more accurate picture of its evolution at the end of the rarefaction curve.
(DOC)

Table S2 Frequencies of taxonomic grouped sequences in each dataset. For each dataset the following data are provided: total number of sequences remaining after quality filtering and removal of chimeras and sequences that are too short (N), number of sequences identified as being of eukaryotic origin (Eukaryota), number of sequences identified as being of bacterial origin (Bacteria), number of sequences that could not truly be assigned (Unclassified) and proportion of unclassified sequences relative to the total number of sequences (U/N).
(DOC)
