# Supplementary material for: Analysis of Metagenomic Data Containing High Biodiversity Levels
Source: PLoS One. 2013 Mar 7;8(3):e58118. doi: 10.1371/journal.pone.0058118 (PMC3591453; doi:10.1371/journal.pone.0058118)
Supplement: Figure S1 — Analysis of synthetic data. The data collected from all the synthetic datasets is summarized as rarefaction curves for observed OTUs at 3% dissimilarity, corrected Chao1 and ACE. Each row in the file contains the results for a given population and its duplicated and triplicated derivatives. The first two rows display the results obtained for the simplistic 16S and V3V5 populations, subsequent rows display the results for all VAMPS-derived populations arranged first by reference database (refV3, refV3V5, refV4V6, refV6A, refV6, refV9 and refSSU) and then by log-normal distribution parameters, from lower to higher σ. Yellow: observed OTUs average, red: Chao1 average, blue: ACE average, as a function of sample size. (PDF) [file pone.0058118.s001.pdf]

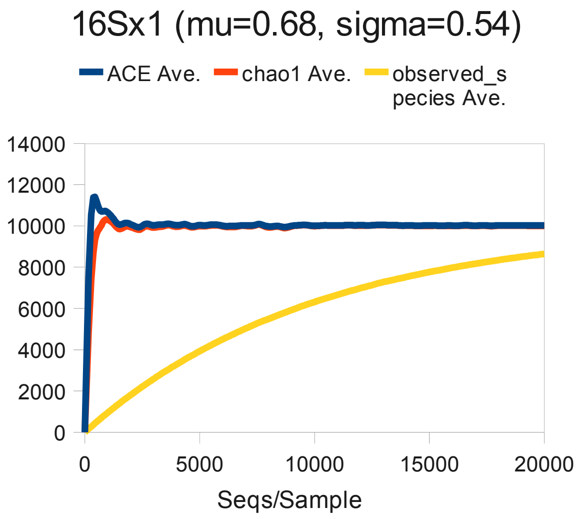
16Sx1 (mu=0.68, sigma=0.54)
ACE Ave.
chao1 Ave.
observed_species Ave.
14000
12000
10000
8000
6000
4000
2000
0
0
5000
10000
15000
20000
Seqs/Sample

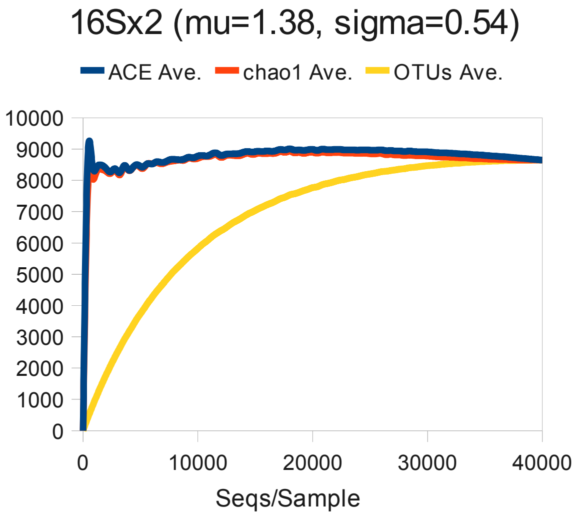
16Sx2 (mu=1.38, sigma=0.54)
ACE Ave.
chao1 Ave.
OTUs Ave.
10000
9000
8000
7000
6000
5000
4000
3000
2000
1000
0
0
10000
20000
30000
40000
Seqs/Sample

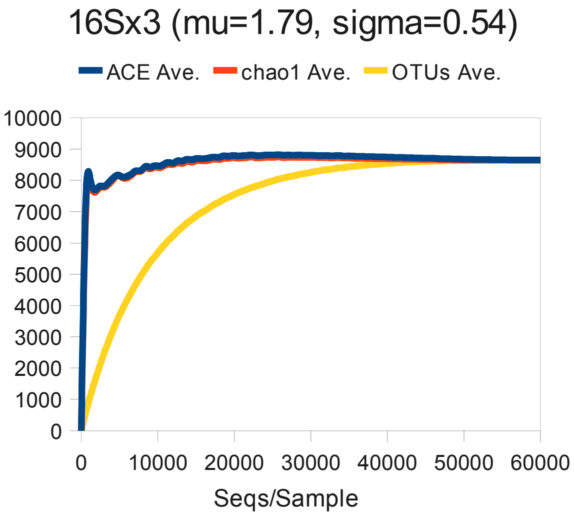
16Sx3 (mu=1.79, sigma=0.54)
ACE Ave.
chao1 Ave.
OTUs Ave.
10000
9000
8000
7000
6000
5000
4000
3000
2000
1000
0
0
10000
20000
30000
40000
50000
60000
Seqs/Sample

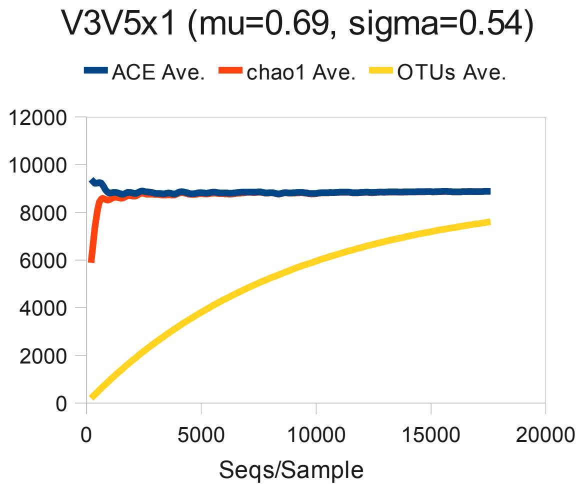
V3V5x1 (mu=0.69, sigma=0.54)
ACE Ave.
chao1 Ave.
OTUs Ave.
12000
10000
8000
6000
4000
2000
0
0
5000
10000
15000
20000
Seqs/Sample

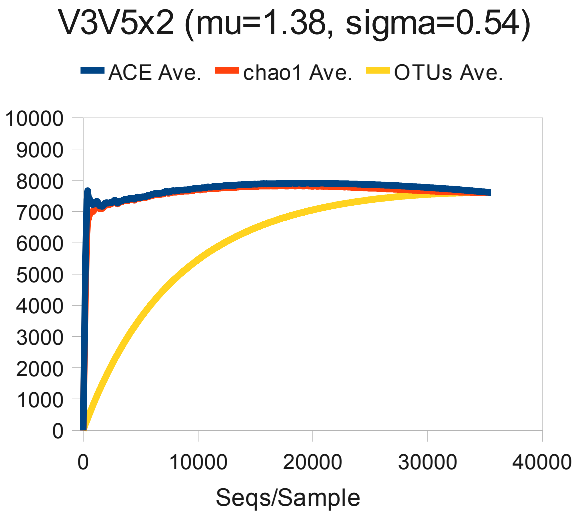
V3V5x2 (mu=1.38, sigma=0.54)
ACE Ave.
chao1 Ave.
OTUs Ave.
10000
9000
8000
7000
6000
5000
4000
3000
2000
1000
0
0
10000
20000
30000
40000
Seqs/Sample

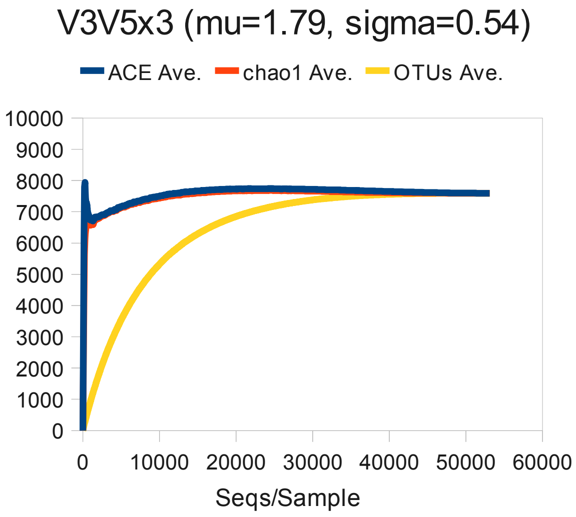
V3V5x3 (mu=1.79, sigma=0.54)
ACE Ave.
chao1 Ave.
OTUs Ave.
10000
9000
8000
7000
6000
5000
4000
3000
2000
1000
0
0
10000
20000
30000
40000
50000
60000
Seqs/Sample

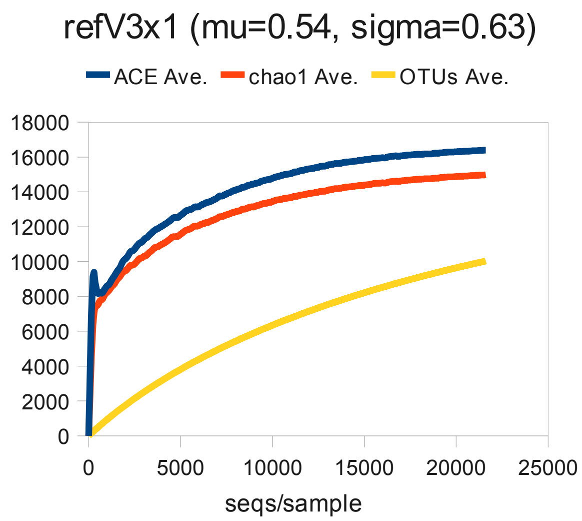
refV3x1 (mu=0.54, sigma=0.63)
ACE Ave.
chao1 Ave.
OTUs Ave.
seqs/sample

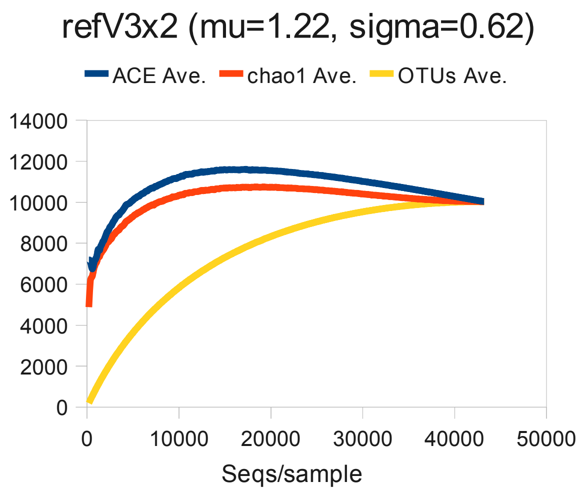
refV3x2 (mu=1.22, sigma=0.62)
ACE Ave.
chao1 Ave.
OTUs Ave.
Seqs/sample

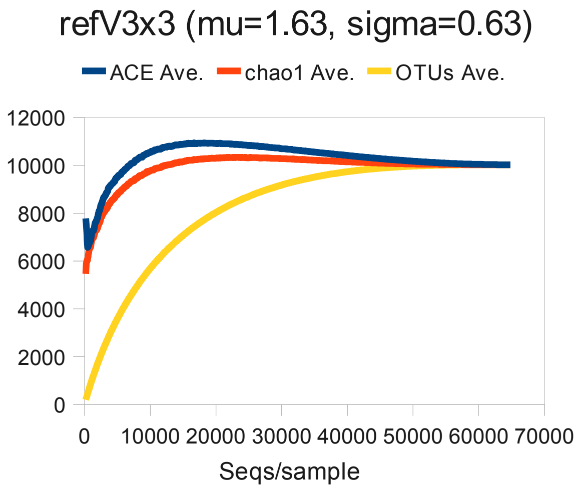
refV3x3 (mu=1.63, sigma=0.63)
ACE Ave.
chao1 Ave.
OTUs Ave.
Seqs/sample

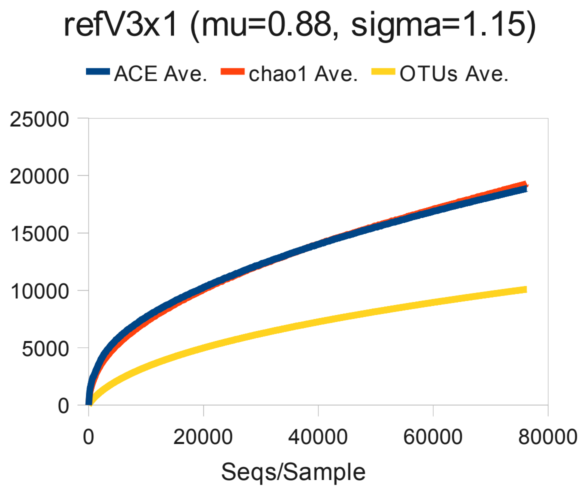
refV3x1 (mu=0.88, sigma=1.15)
ACE Ave.
chao1 Ave.
OTUs Ave.
Seqs/Sample

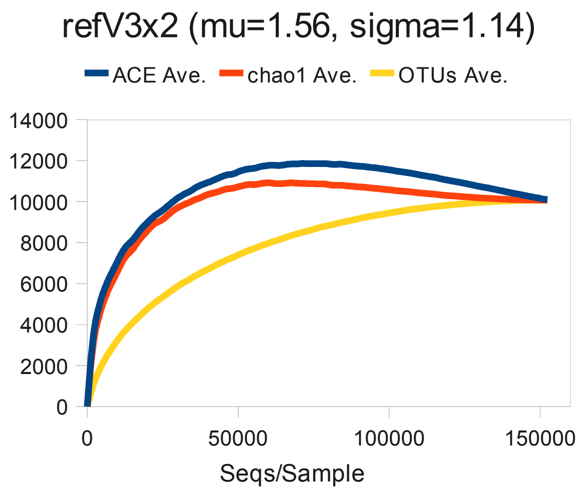
refV3x2 (mu=1.56, sigma=1.14)
ACE Ave.
chao1 Ave.
OTUs Ave.
Seqs/Sample

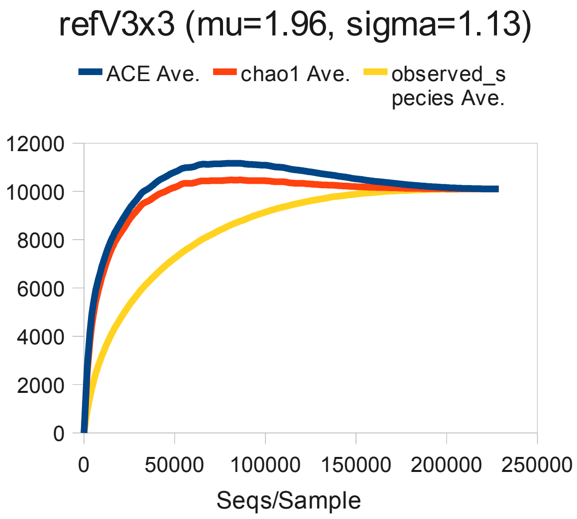
refV3x3 (mu=1.96, sigma=1.13)
ACE Ave.
chao1 Ave.
observed_species Ave.
Seqs/Sample

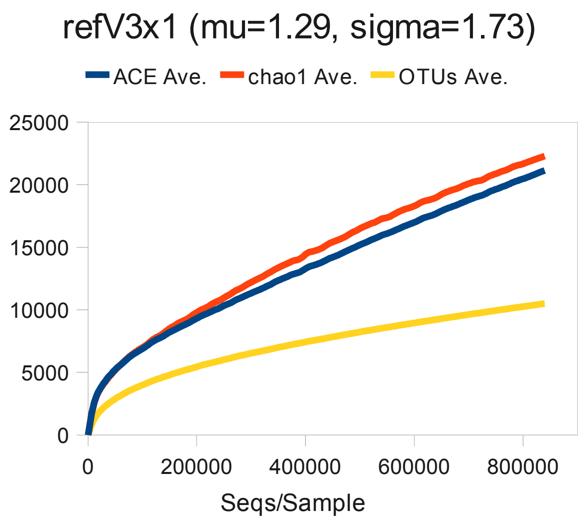
refV3x1 (mu=1.29, sigma=1.73)
ACE Ave. chao1 Ave. OTUs Ave.
25000
20000
15000
10000
5000
0
0 200000 400000 600000 800000
Seqs/Sample

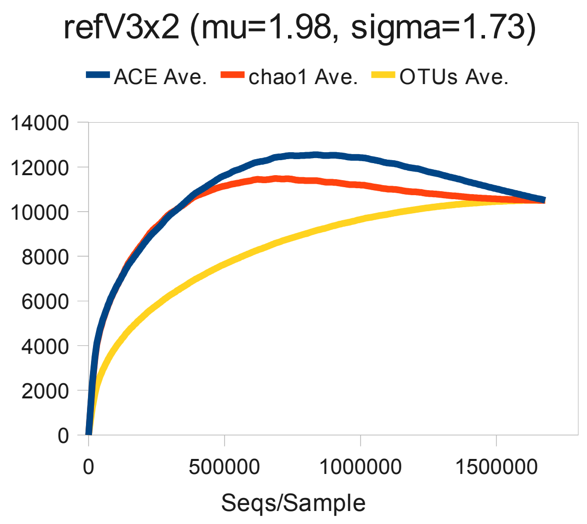
refV3x2 (mu=1.98, sigma=1.73)
ACE Ave. chao1 Ave. OTUs Ave.
14000
12000
10000
8000
6000
4000
2000
0
0 500000 1000000 1500000
Seqs/Sample

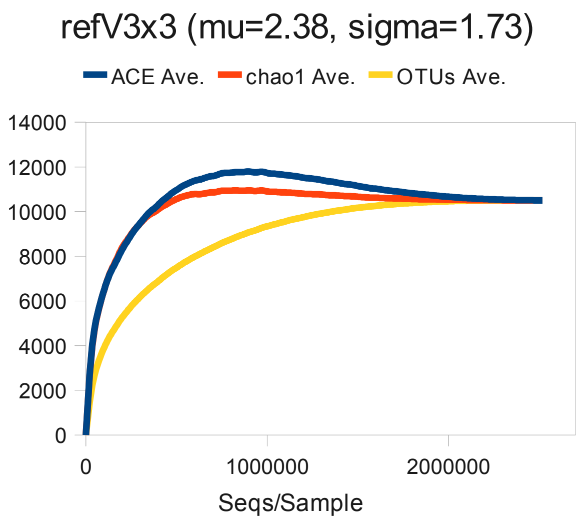
refV3x3 (mu=2.38, sigma=1.73)
ACE Ave. chao1 Ave. OTUs Ave.
14000
12000
10000
8000
6000
4000
2000
0
0 1000000 2000000
Seqs/Sample

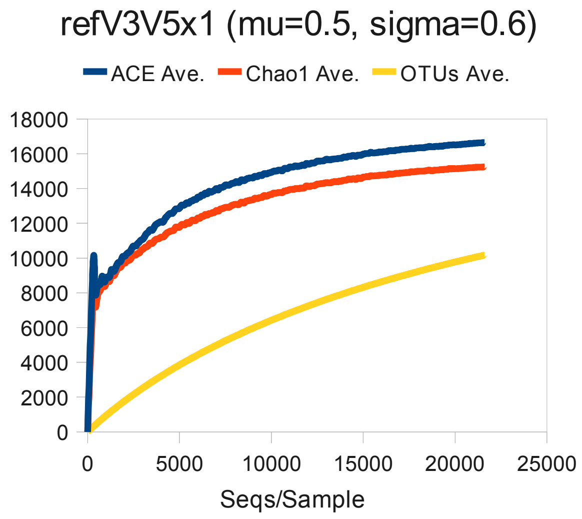
refV3V5x1 (mu=0.5, sigma=0.6)
ACE Ave. Chao1 Ave. OTUs Ave.
18000
16000
14000
12000
10000
8000
6000
4000
2000
0
0 5000 10000 15000 20000 25000
Seqs/Sample

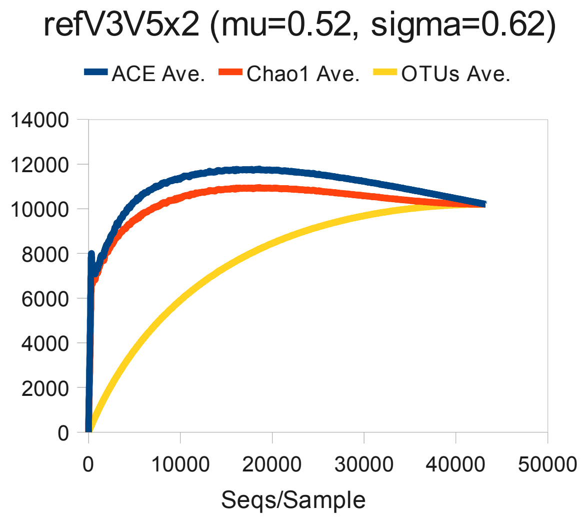
refV3V5x2 (mu=0.52, sigma=0.62)
ACE Ave. Chao1 Ave. OTUs Ave.
14000
12000
10000
8000
6000
4000
2000
0
0 10000 20000 30000 40000 50000
Seqs/Sample

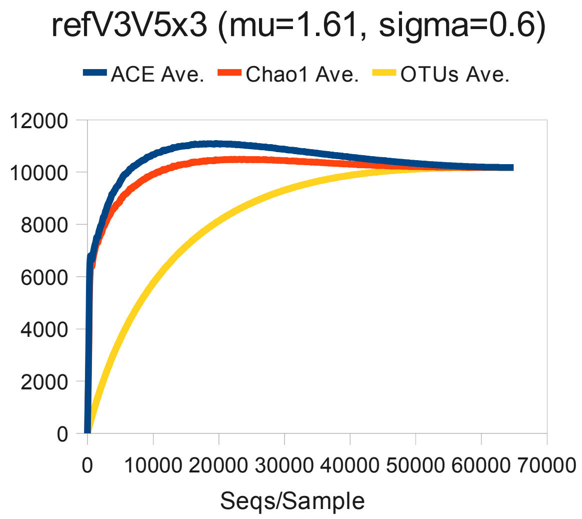
refV3V5x3 (mu=1.61, sigma=0.6)
ACE Ave. Chao1 Ave. OTUs Ave.
12000
10000
8000
6000
4000
2000
0
0 10000 20000 30000 40000 50000 60000 70000
Seqs/Sample

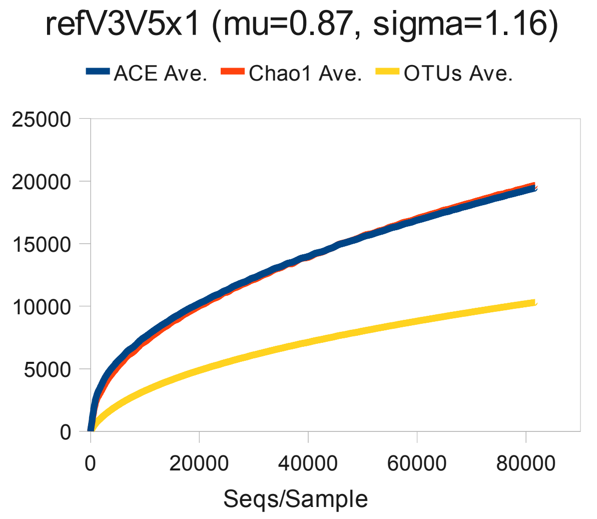
refV3V5x1 (mu=0.87, sigma=1.16)
ACE Ave.
Chao1 Ave.
OTUs Ave.
25000
20000
15000
10000
5000
0
0
20000
40000
60000
80000
Seqs/Sample

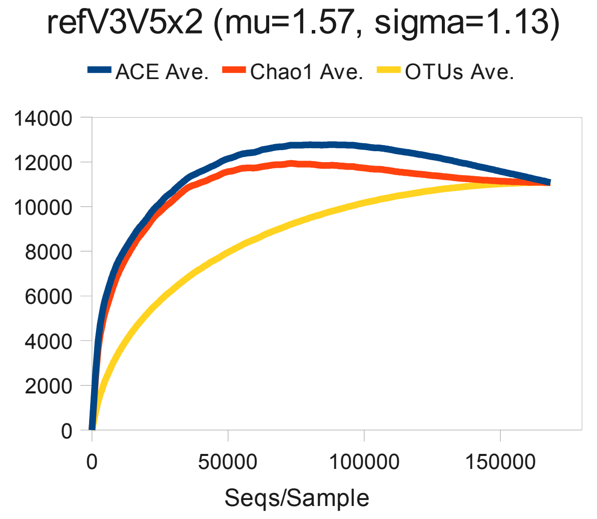
refV3V5x2 (mu=1.57, sigma=1.13)
ACE Ave.
Chao1 Ave.
OTUs Ave.
14000
12000
10000
8000
6000
4000
2000
0
0
50000
100000
150000
Seqs/Sample

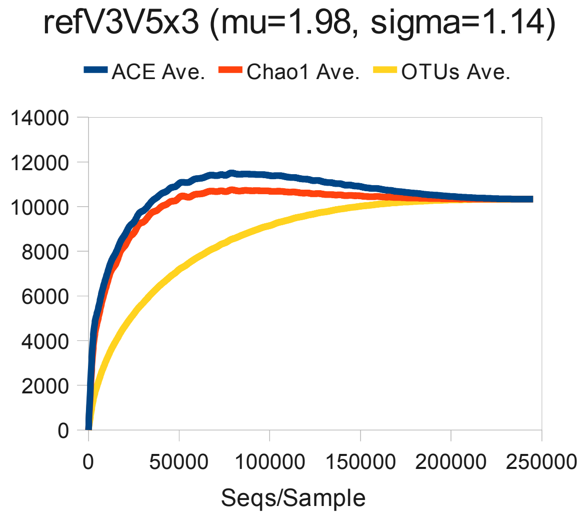
refV3V5x3 (mu=1.98, sigma=1.14)
ACE Ave.
Chao1 Ave.
OTUs Ave.
14000
12000
10000
8000
6000
4000
2000
0
0
50000
100000
150000
200000
250000
Seqs/Sample

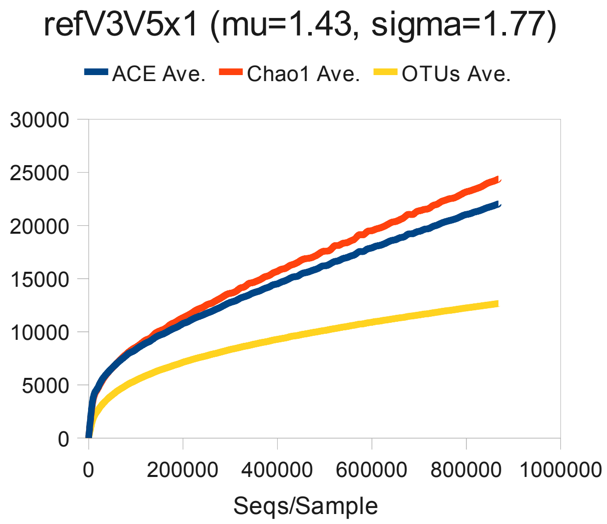
refV3V5x1 (mu=1.43, sigma=1.77)
ACE Ave.
Chao1 Ave.
OTUs Ave.
30000
25000
20000
15000
10000
5000
0
0
200000
400000
600000
800000
1000000
Seqs/Sample

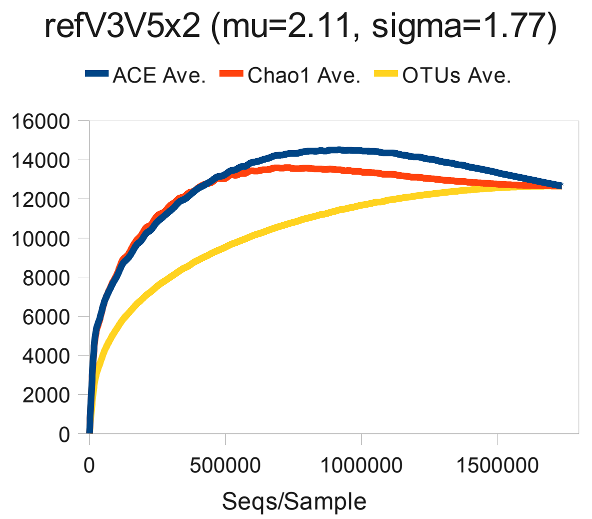
refV3V5x2 (mu=2.11, sigma=1.77)
ACE Ave.
Chao1 Ave.
OTUs Ave.
16000
14000
12000
10000
8000
6000
4000
2000
0
0
500000
1000000
1500000
Seqs/Sample

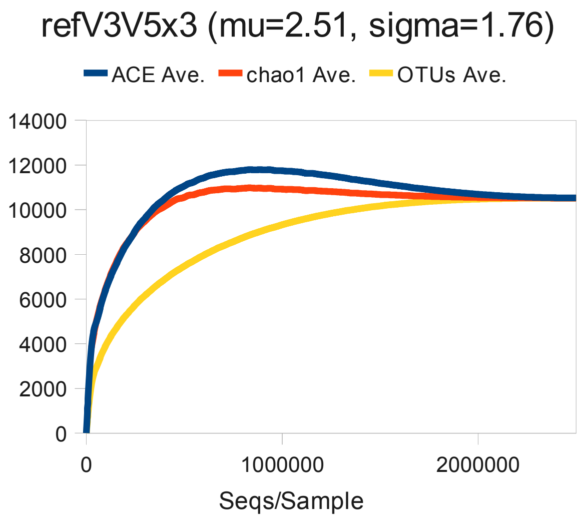
refV3V5x3 (mu=2.51, sigma=1.76)
ACE Ave.
chao1 Ave.
OTUs Ave.
14000
12000
10000
8000
6000
4000
2000
0
0
1000000
2000000
Seqs/Sample

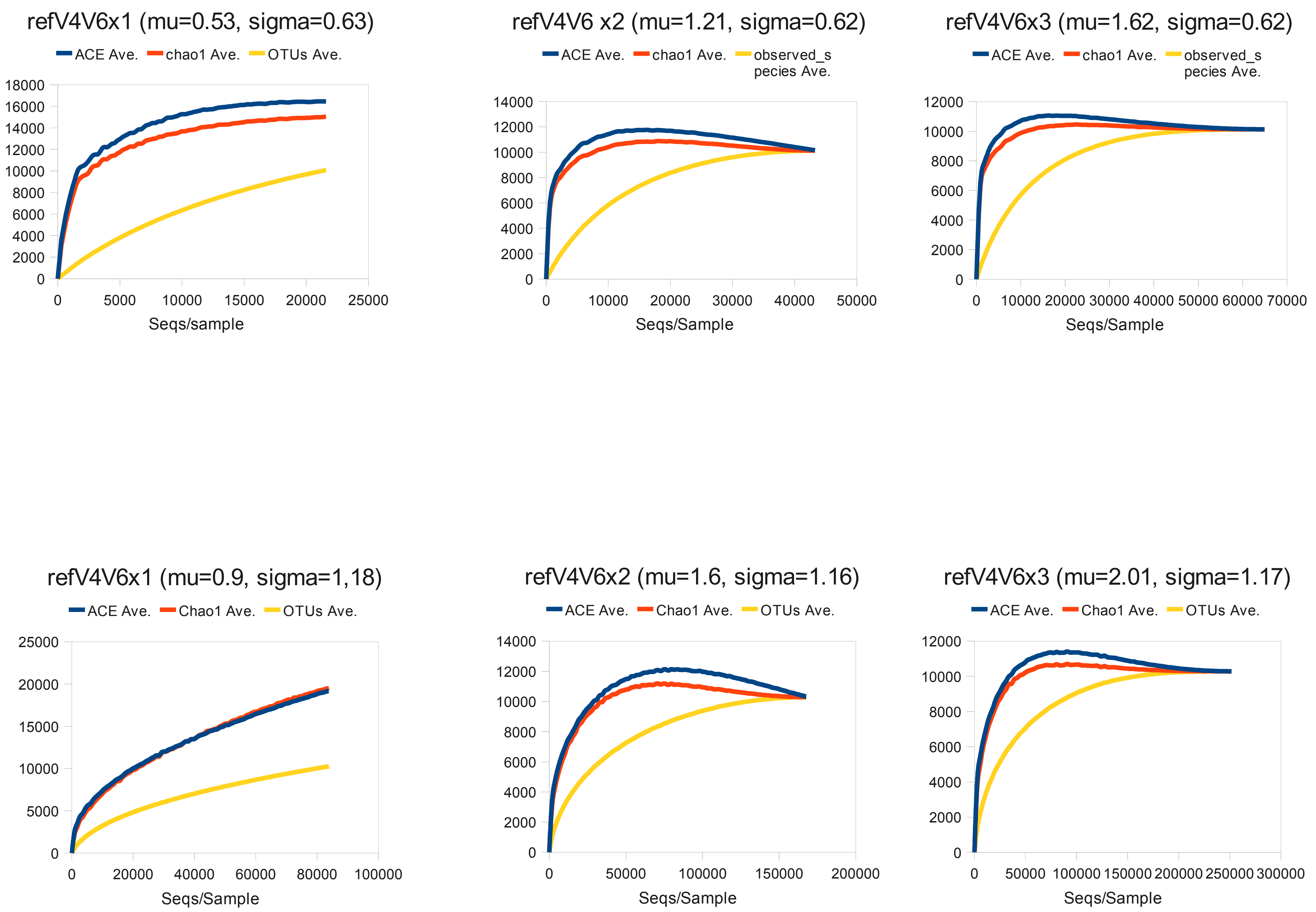
refV4V6x1 (mu=0.53, sigma=0.63)
ACE Ave.
chao1 Ave.
OTUs Ave.
18000
16000
14000
12000
10000
8000
6000
4000
2000
0
0
5000
10000
15000
20000
25000
Seqs/sample
refV4V6 x2 (mu=1.21, sigma=0.62)
ACE Ave.
chao1 Ave.
observed_species Ave.
14000
12000
10000
8000
6000
4000
2000
0
0
10000
20000
30000
40000
50000
Seqs/Sample
refV4V6x3 (mu=1.62, sigma=0.62)
ACE Ave.
chao1 Ave.
observed_species Ave.
12000
10000
8000
6000
4000
2000
0
0
10000
20000
30000
40000
50000
60000
70000
Seqs/Sample
refV4V6x1 (mu=0.9, sigma=1,18)
ACE Ave.
Chao1 Ave.
OTUs Ave.
25000
20000
15000
10000
5000
0
0
20000
40000
60000
80000
100000
Seqs/Sample
refV4V6x2 (mu=1.6, sigma=1.16)
ACE Ave.
Chao1 Ave.
OTUs Ave.
14000
12000
10000
8000
6000
4000
2000
0
0
50000
100000
150000
200000
Seqs/Sample
refV4V6x3 (mu=2.01, sigma=1.17)
ACE Ave.
Chao1 Ave.
OTUs Ave.
12000
10000
8000
6000
4000
2000
0
0
50000
100000
150000
200000
250000
300000
Seqs/Sample

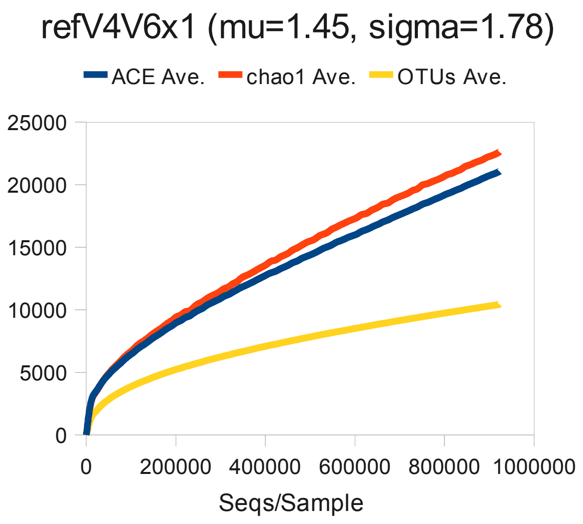
refV4V6x1 (mu=1.45, sigma=1.78)
ACE Ave.
chao1 Ave.
OTUs Ave.
25000
20000
15000
10000
5000
0
0
200000
400000
600000
800000
1000000
Seqs/Sample

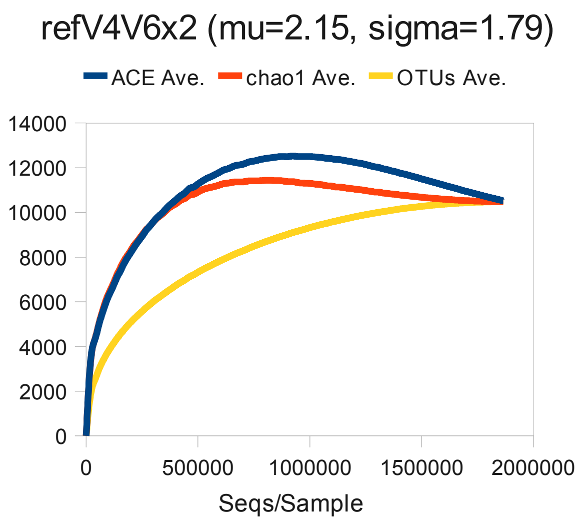
refV4V6x2 (mu=2.15, sigma=1.79)
ACE Ave.
chao1 Ave.
OTUs Ave.
14000
12000
10000
8000
6000
4000
2000
0
0
500000
1000000
1500000
2000000
Seqs/Sample

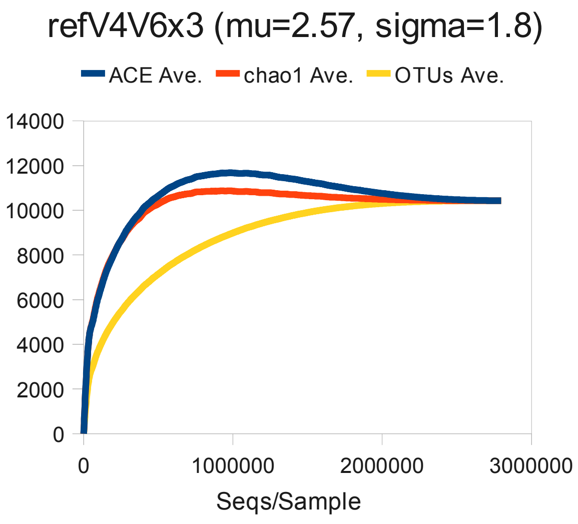
refV4V6x3 (mu=2.57, sigma=1.8)
ACE Ave.
chao1 Ave.
OTUs Ave.
14000
12000
10000
8000
6000
4000
2000
0
0
1000000
2000000
3000000
Seqs/Sample

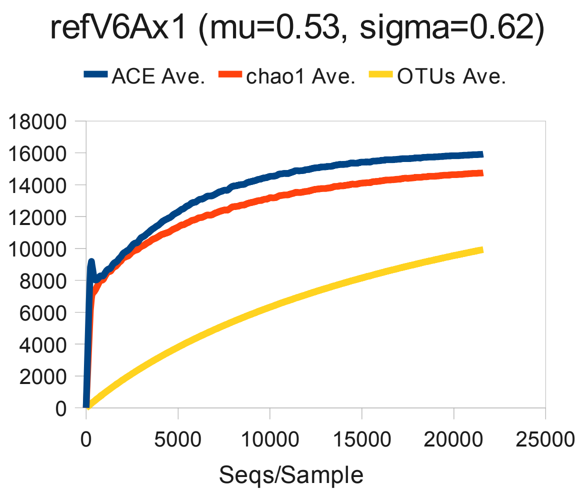
refV6Ax1 (mu=0.53, sigma=0.62)
ACE Ave.
chao1 Ave.
OTUs Ave.
18000
16000
14000
12000
10000
8000
6000
4000
2000
0
0
5000
10000
15000
20000
25000
Seqs/Sample

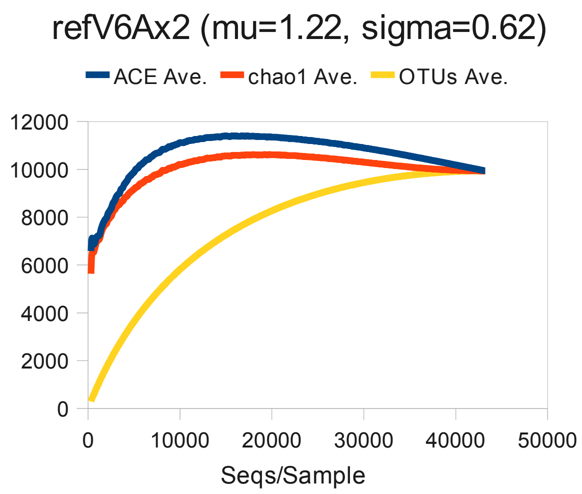
refV6Ax2 (mu=1.22, sigma=0.62)
ACE Ave.
chao1 Ave.
OTUs Ave.
12000
10000
8000
6000
4000
2000
0
0
10000
20000
30000
40000
50000
Seqs/Sample

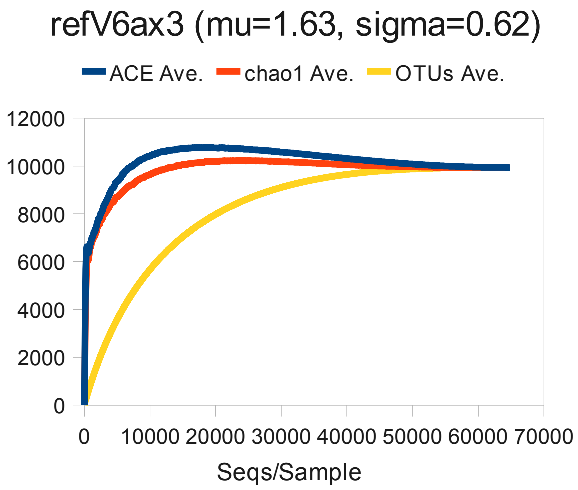
refV6ax3 (mu=1.63, sigma=0.62)
ACE Ave.
chao1 Ave.
OTUs Ave.
12000
10000
8000
6000
4000
2000
0
0
10000
20000
30000
40000
50000
60000
70000
Seqs/Sample

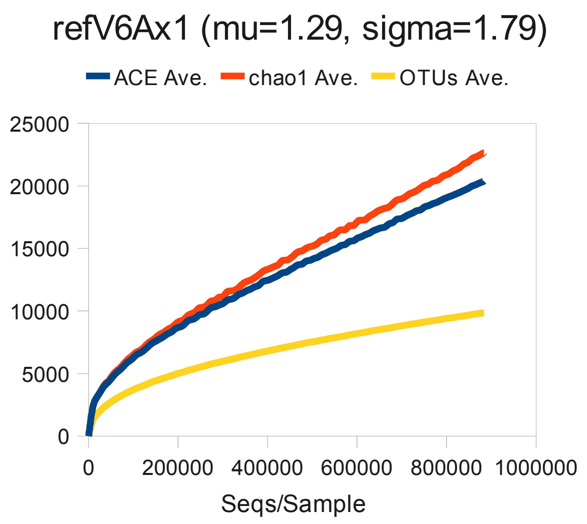
refV6Ax1 (mu=1.29, sigma=1.79)
ACE Ave.
chao1 Ave.
OTUs Ave.
25000
20000
15000
10000
5000
0
0
200000
400000
600000
800000
1000000
Seqs/Sample

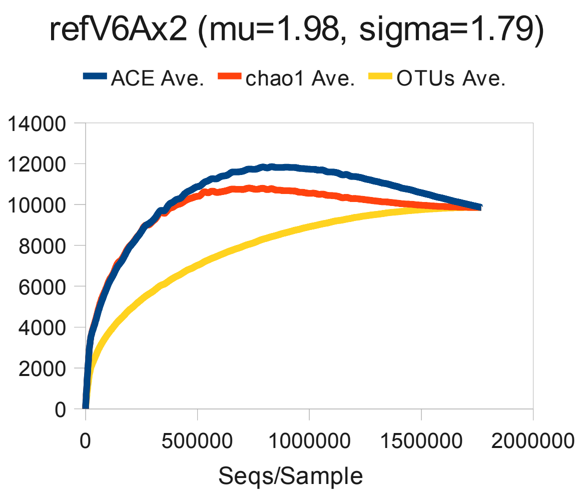
refV6Ax2 (mu=1.98, sigma=1.79)
ACE Ave.
chao1 Ave.
OTUs Ave.
14000
12000
10000
8000
6000
4000
2000
0
0
500000
1000000
1500000
2000000
Seqs/Sample

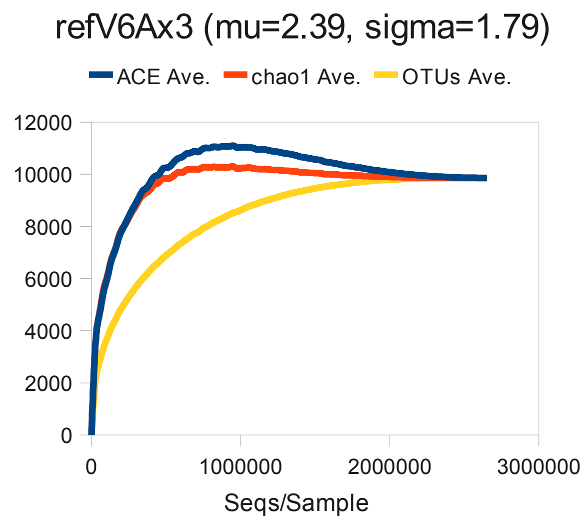
refV6Ax3 (mu=2.39, sigma=1.79)
ACE Ave.
chao1 Ave.
OTUs Ave.
12000
10000
8000
6000
4000
2000
0
0
1000000
2000000
3000000
Seqs/Sample

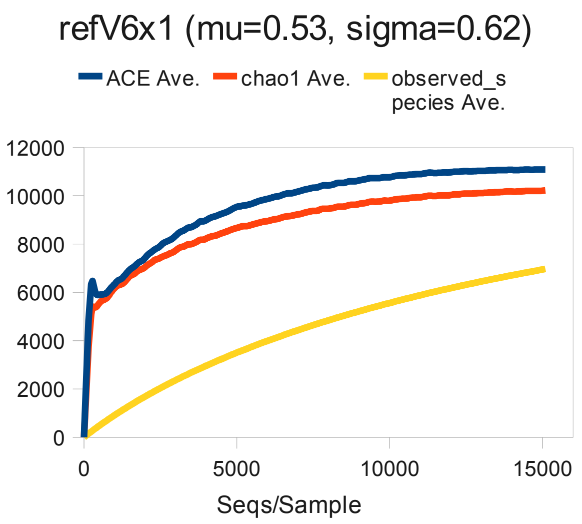
refV6x1 (mu=0.53, sigma=0.62)
ACE Ave.
chao1 Ave.
observed_s pecies Ave.
12000
10000
8000
6000
4000
2000
0
0
5000
10000
15000
Seqs/Sample

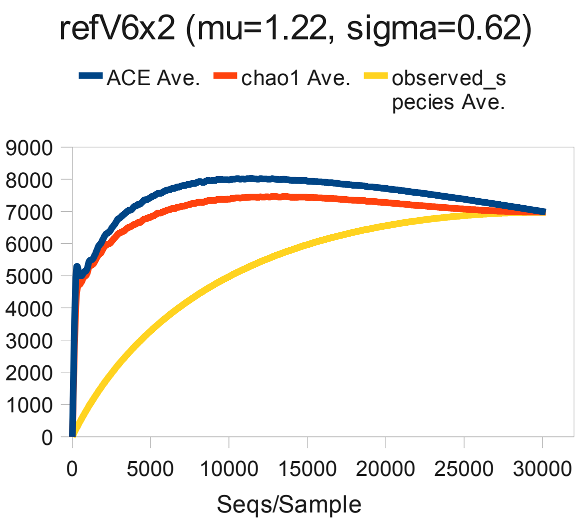
refV6x2 (mu=1.22, sigma=0.62)
ACE Ave.
chao1 Ave.
observed_s pecies Ave.
9000
8000
7000
6000
5000
4000
3000
2000
1000
0
0
5000
10000
15000
20000
25000
30000
Seqs/Sample

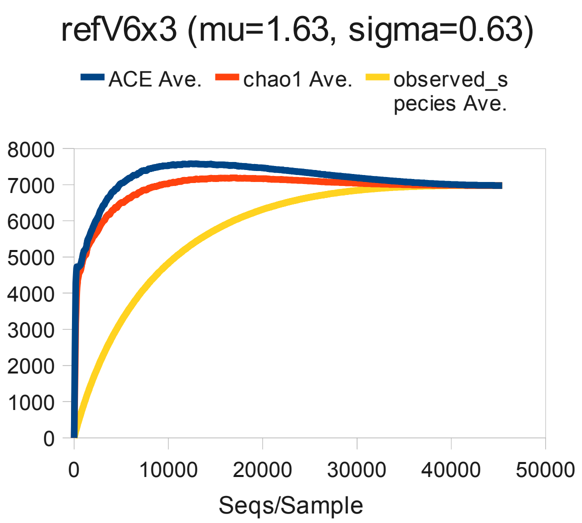
refV6x3 (mu=1.63, sigma=0.63)
ACE Ave.
chao1 Ave.
observed_s pecies Ave.
8000
7000
6000
5000
4000
3000
2000
1000
0
0
10000
20000
30000
40000
50000
Seqs/Sample

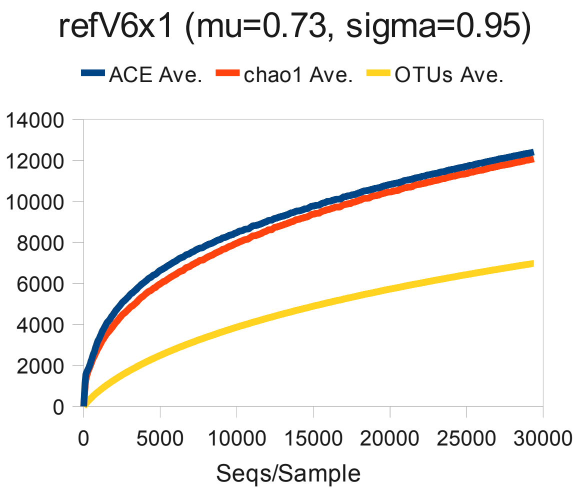
refV6x1 (mu=0.73, sigma=0.95)
ACE Ave.
chao1 Ave.
OTUs Ave.
14000
12000
10000
8000
6000
4000
2000
0
0
5000
10000
15000
20000
25000
30000
Seqs/Sample

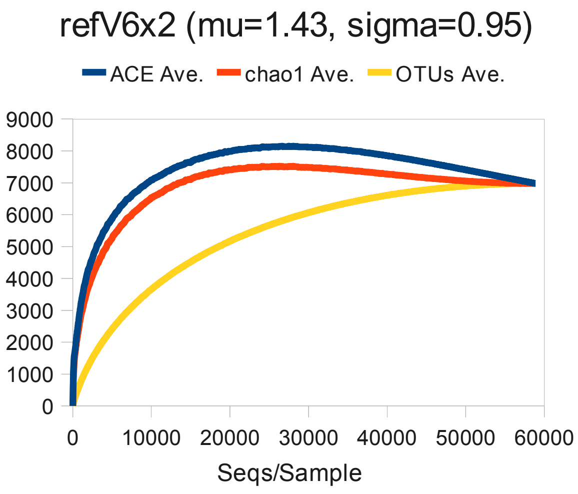
refV6x2 (mu=1.43, sigma=0.95)
ACE Ave.
chao1 Ave.
OTUs Ave.
9000
8000
7000
6000
5000
4000
3000
2000
1000
0
0
10000
20000
30000
40000
50000
60000
Seqs/Sample

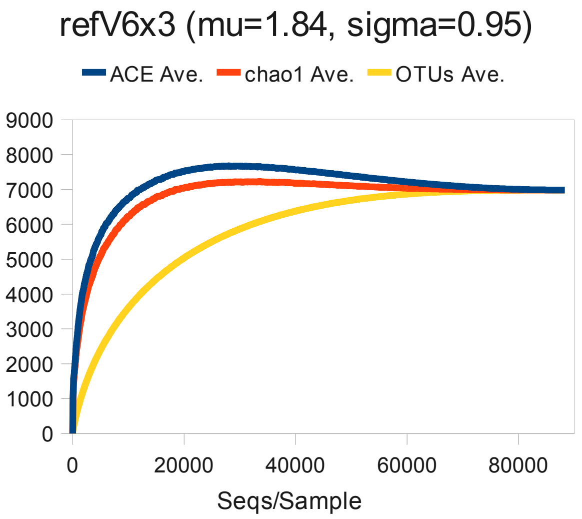
refV6x3 (mu=1.84, sigma=0.95)
ACE Ave.
chao1 Ave.
OTUs Ave.
9000
8000
7000
6000
5000
4000
3000
2000
1000
0
0
20000
40000
60000
80000
Seqs/Sample

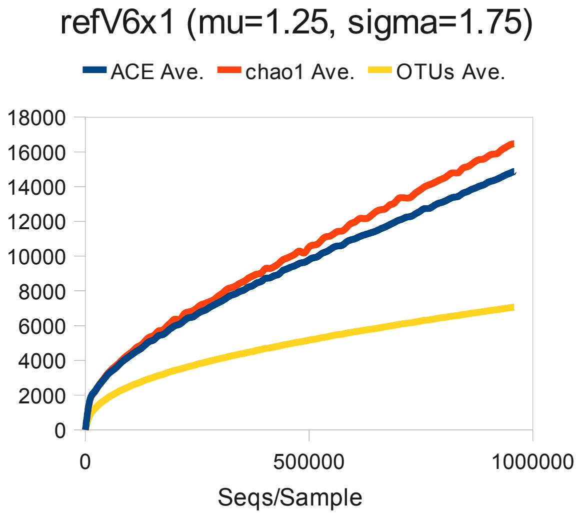
refV6x1 (mu=1.25, sigma=1.75)
ACE Ave.
chao1 Ave.
OTUs Ave.
18000
16000
14000
12000
10000
8000
6000
4000
2000
0
0
500000
1000000
Seqs/Sample

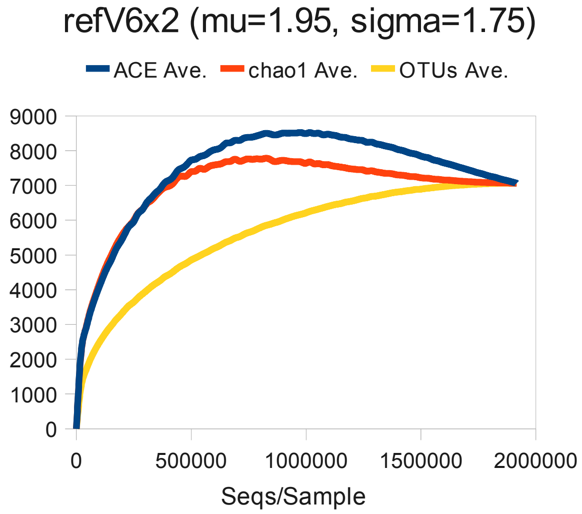
refV6x2 (mu=1.95, sigma=1.75)
ACE Ave.
chao1 Ave.
OTUs Ave.
9000
8000
7000
6000
5000
4000
3000
2000
1000
0
0
500000
1000000
1500000
2000000
Seqs/Sample

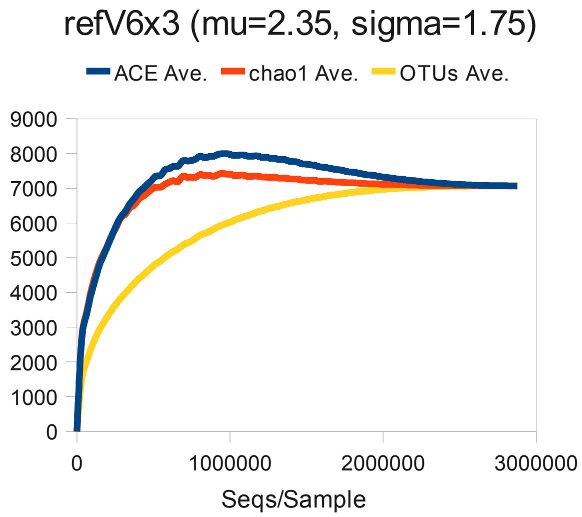
refV6x3 (mu=2.35, sigma=1.75)
ACE Ave.
chao1 Ave.
OTUs Ave.
9000
8000
7000
6000
5000
4000
3000
2000
1000
0
0
1000000
2000000
3000000
Seqs/Sample

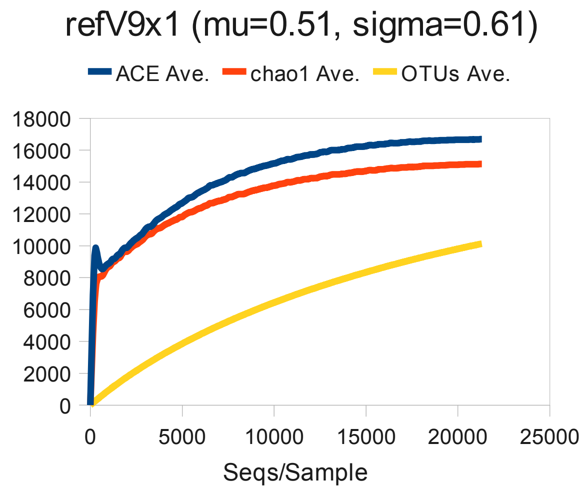
refV9x1 (mu=0.51, sigma=0.61)
ACE Ave.
chao1 Ave.
OTUs Ave.
18000
16000
14000
12000
10000
8000
6000
4000
2000
0
0
5000
10000
15000
20000
25000
Seqs/Sample

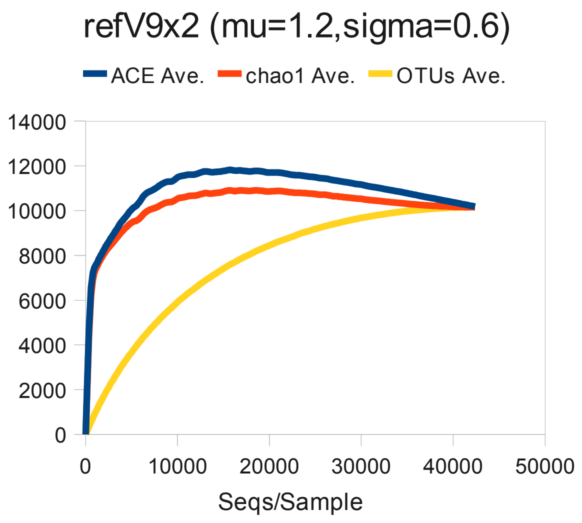
refV9x2 (mu=1.2,sigma=0.6)
ACE Ave.
chao1 Ave.
OTUs Ave.
14000
12000
10000
8000
6000
4000
2000
0
0
10000
20000
30000
40000
50000
Seqs/Sample

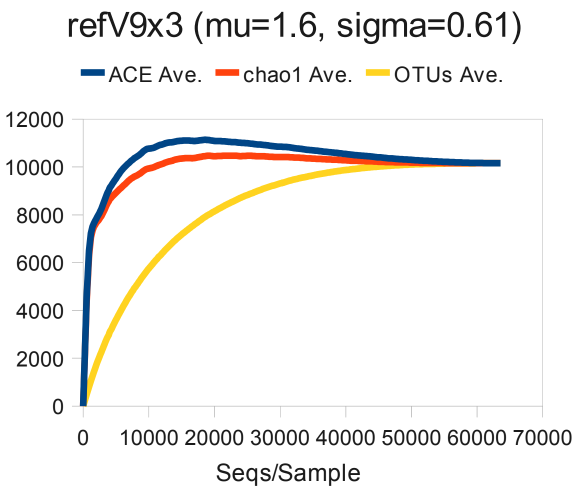
refV9x3 (mu=1.6, sigma=0.61)
ACE Ave.
chao1 Ave.
OTUs Ave.
12000
10000
8000
6000
4000
2000
0
0
10000 20000 30000 40000 50000 60000 70000
Seqs/Sample

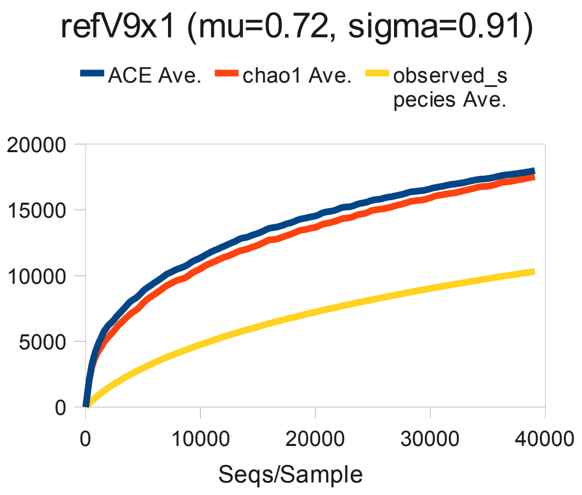
refV9x1 (mu=0.72, sigma=0.91)
ACE Ave.
chao1 Ave.
observed_s pecies Ave.
20000
15000
10000
5000
0
0
10000
20000
30000
40000
Seqs/Sample

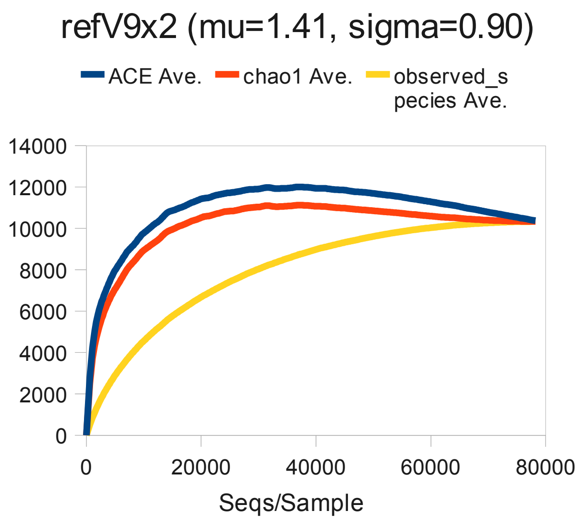
refV9x2 (mu=1.41, sigma=0.90)
ACE Ave.
chao1 Ave.
observed_s pecies Ave.
14000
12000
10000
8000
6000
4000
2000
0
0
20000
40000
60000
80000
Seqs/Sample

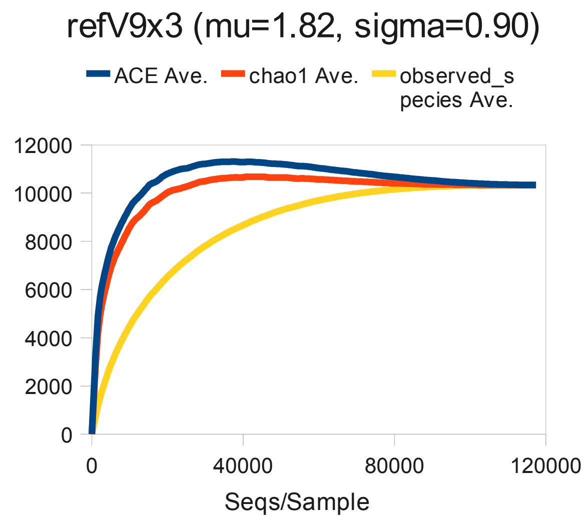
refV9x3 (mu=1.82, sigma=0.90)
ACE Ave.
chao1 Ave.
observed_s pecies Ave.
12000
10000
8000
6000
4000
2000
0
0
40000
80000
120000
Seqs/Sample

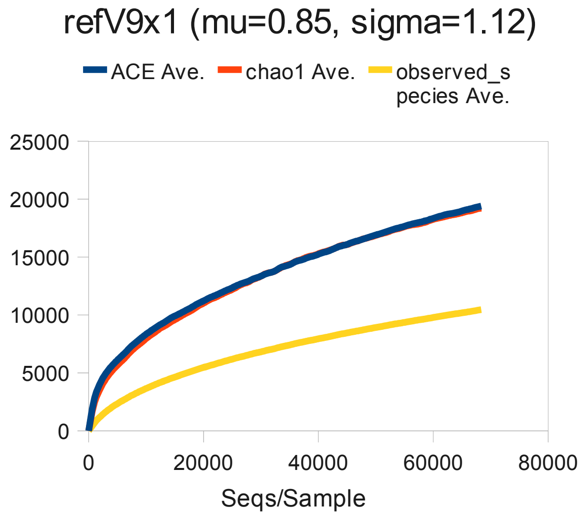
refV9x1 (mu=0.85, sigma=1.12)
ACE Ave.
chao1 Ave.
observed_species Ave.
0
5000
10000
15000
20000
25000
0
20000
40000
60000
80000
Seqs/Sample

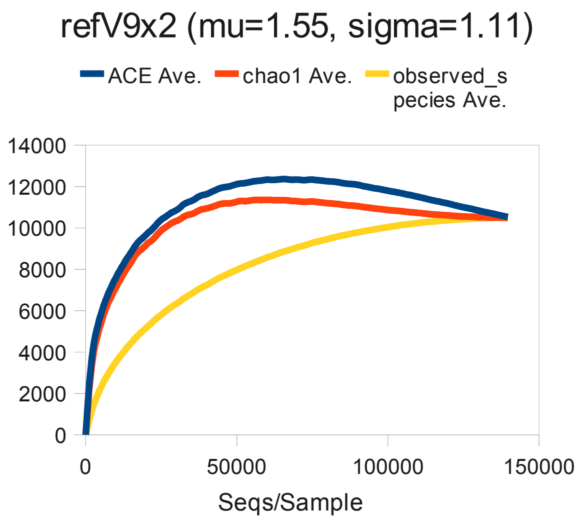
refV9x2 (mu=1.55, sigma=1.11)
ACE Ave.
chao1 Ave.
observed_species Ave.
0
2000
4000
6000
8000
10000
12000
14000
0
50000
100000
150000
Seqs/Sample

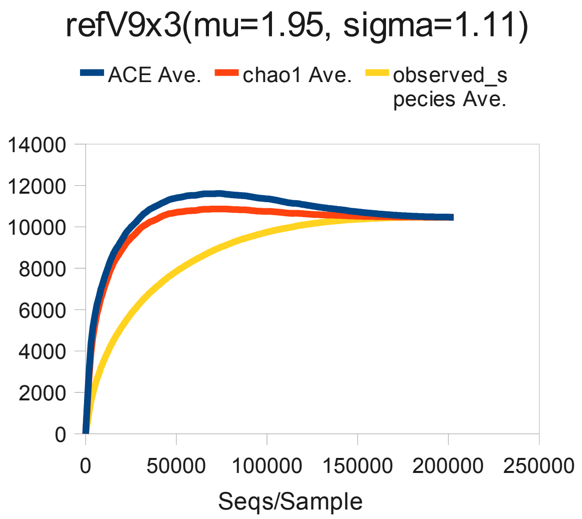
refV9x3(mu=1.95, sigma=1.11)
ACE Ave.
chao1 Ave.
observed_species Ave.
0
2000
4000
6000
8000
10000
12000
14000
0
50000
100000
150000
200000
250000
Seqs/Sample

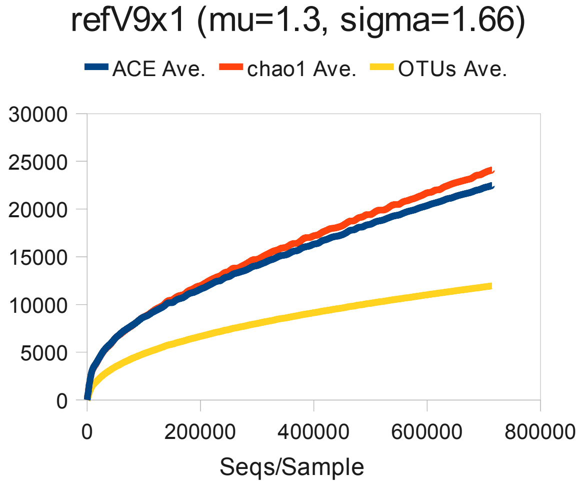
refV9x1 (mu=1.3, sigma=1.66)
ACE Ave.
chao1 Ave.
OTUs Ave.
0
5000
10000
15000
20000
25000
30000
0
200000
400000
600000
800000
Seqs/Sample

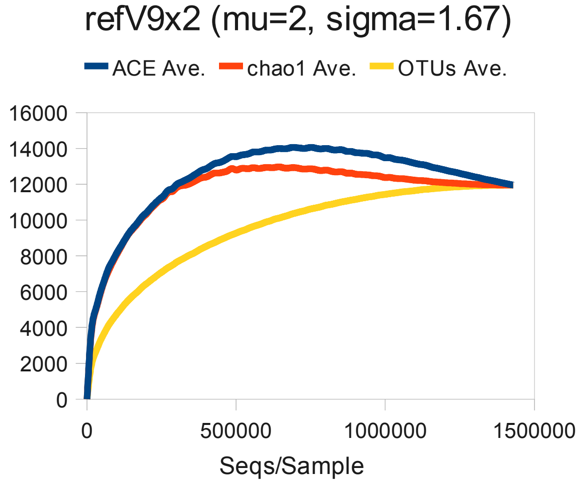
refV9x2 (mu=2, sigma=1.67)
ACE Ave.
chao1 Ave.
OTUs Ave.
0
2000
4000
6000
8000
10000
12000
14000
16000
0
500000
1000000
1500000
Seqs/Sample

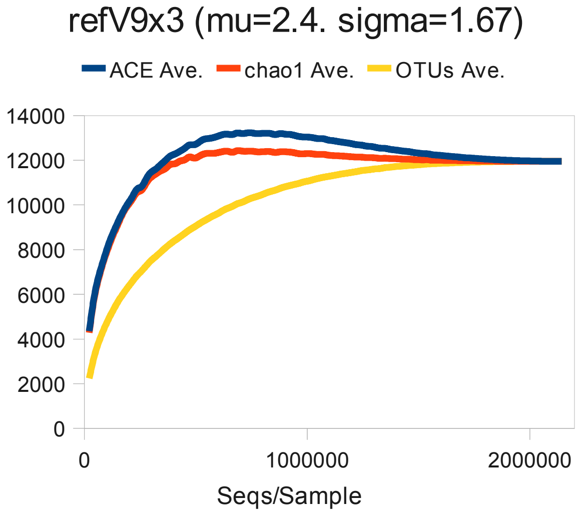
refV9x3 (mu=2.4. sigma=1.67)
ACE Ave.
chao1 Ave.
OTUs Ave.
0
2000
4000
6000
8000
10000
12000
14000
0
1000000
2000000
Seqs/Sample

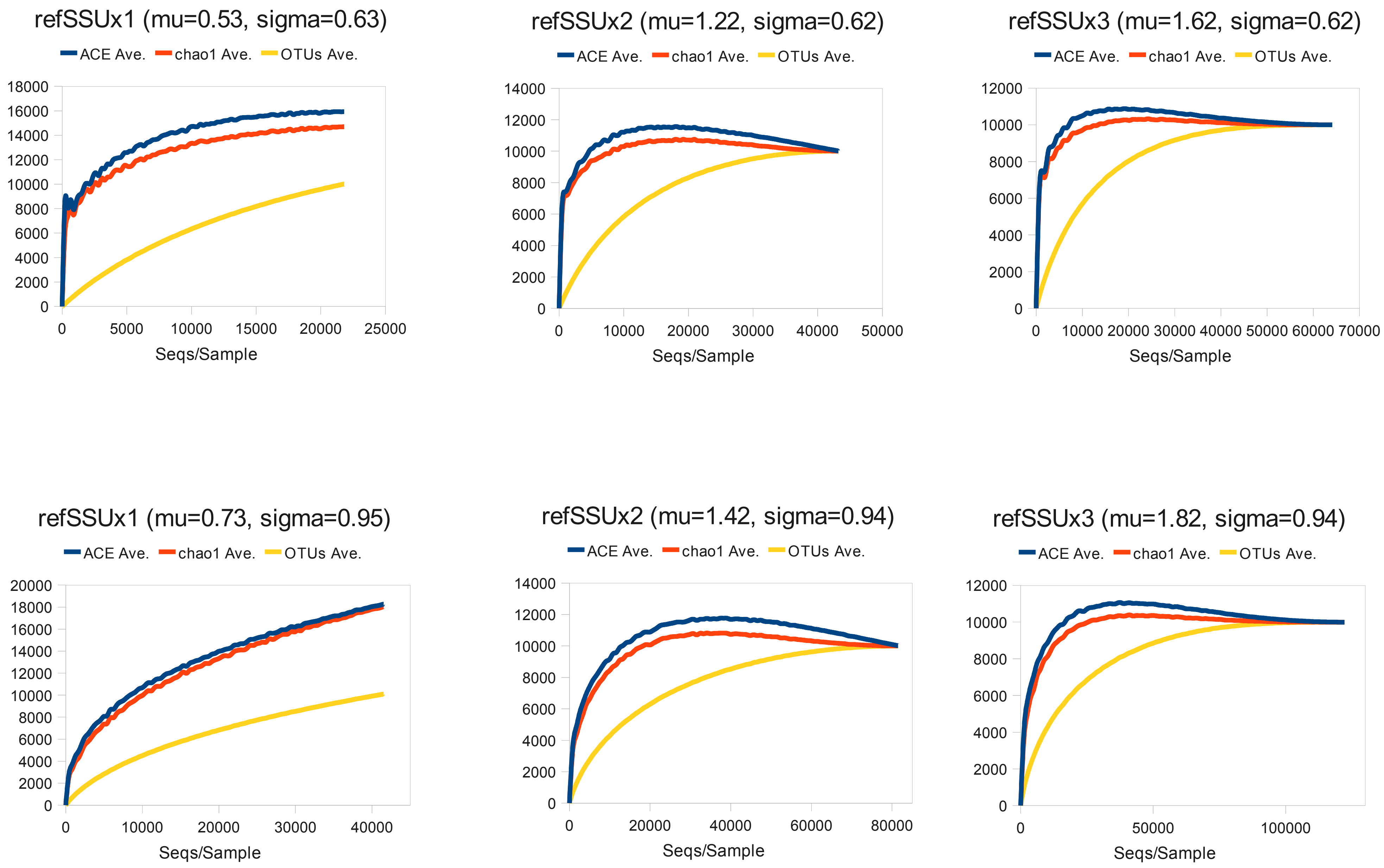
refSSUx1 (mu=0.53, sigma=0.63)
ACE Ave.
chao1 Ave.
OTUs Ave.
0
2000
4000
6000
8000
10000
12000
14000
16000
18000
0
5000
10000
15000
20000
25000
Seqs/Sample
refSSUx2 (mu=1.22, sigma=0.62)
ACE Ave.
chao1 Ave.
OTUs Ave.
0
2000
4000
6000
8000
10000
12000
14000
0
10000
20000
30000
40000
50000
Seqs/Sample
refSSUx3 (mu=1.62, sigma=0.62)
ACE Ave.
chao1 Ave.
OTUs Ave.
0
2000
4000
6000
8000
10000
12000
0
10000
20000
30000
40000
50000
60000
70000
Seqs/Sample
refSSUx1 (mu=0.73, sigma=0.95)
ACE Ave.
chao1 Ave.
OTUs Ave.
0
2000
4000
6000
8000
10000
12000
14000
16000
18000
20000
0
10000
20000
30000
40000
Seqs/Sample
refSSUx2 (mu=1.42, sigma=0.94)
ACE Ave.
chao1 Ave.
OTUs Ave.
0
2000
4000
6000
8000
10000
12000
14000
0
20000
40000
60000
80000
Seqs/Sample
refSSUx3 (mu=1.82, sigma=0.94)
ACE Ave.
chao1 Ave.
OTUs Ave.
0
2000
4000
6000
8000
10000
12000
0
50000
100000
Seqs/Sample

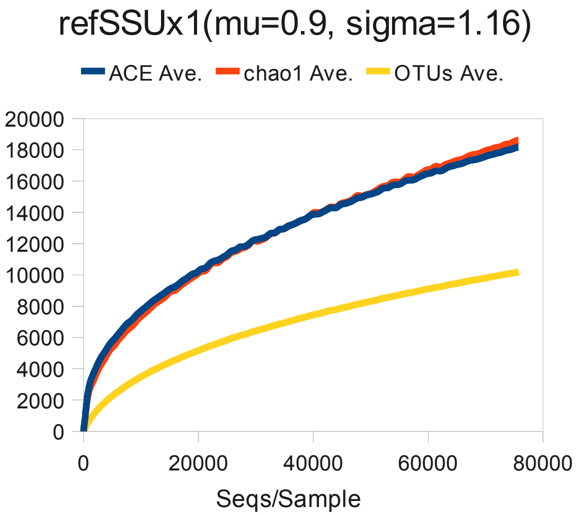
refSSUx1(mu=0.9, sigma=1.16)
ACE Ave.
chao1 Ave.
OTUs Ave.
20000
18000
16000
14000
12000
10000
8000
6000
4000
2000
0
0
20000
40000
60000
80000
Seqs/Sample

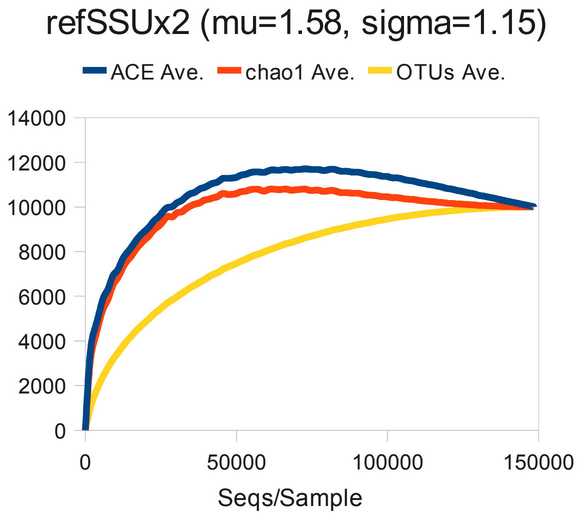
refSSUx2 (mu=1.58, sigma=1.15)
ACE Ave.
chao1 Ave.
OTUs Ave.
14000
12000
10000
8000
6000
4000
2000
0
0
50000
100000
150000
Seqs/Sample

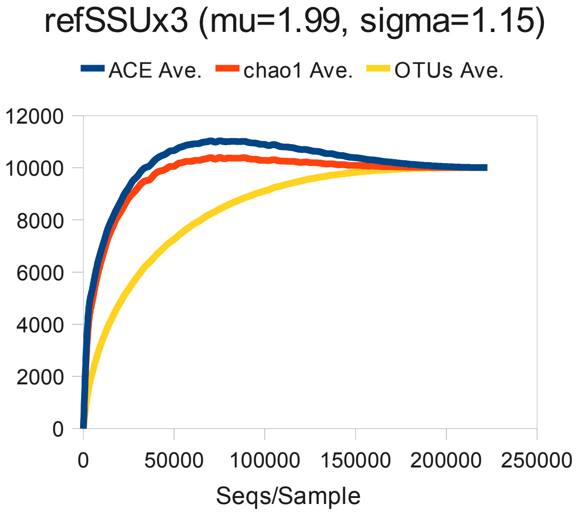
refSSUx3 (mu=1.99, sigma=1.15)
ACE Ave.
chao1 Ave.
OTUs Ave.
12000
10000
8000
6000
4000
2000
0
0
50000
100000
150000
200000
250000
Seqs/Sample
