# Supplementary material for: Analysis of Metagenomic Data Containing High Biodiversity Levels
Source: PLoS One. 2013 Mar 7;8(3):e58118. doi: 10.1371/journal.pone.0058118 (PMC3591453; doi:10.1371/journal.pone.0058118)
Supplement: Figure S2 — Rarefaction curves from experimental data sets. Evolution of Chao1 and OTUs figures with the sample size. The dashed line represents the ACE average, the dotted line represents the Chao1 average and the continuous line the OTUs distribution at 3% dissimilarity. Left column (clean): results obtained after quality filtering and removal of chimeras and short sequences. Right column (-euk -UC1): results obtained after additional removal of eukaryotic sequences and unclassified sequences that clustered as singleton OTUs. Yellow: observed OTUs average, red: Chao1 average, blue: ACE average, at 3% dissimilarity and as a function of sample size. (PDF) [file pone.0058118.s002.pdf]

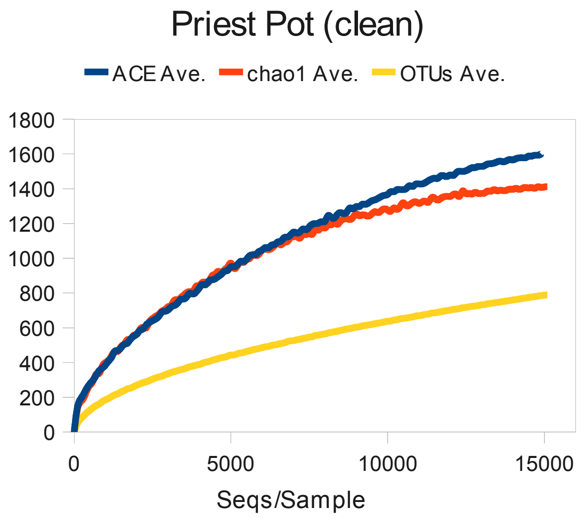
Priest Pot (clean)
ACE Ave.
chao1 Ave.
OTUs Ave.
1800
1600
1400
1200
1000
800
600
400
200
0
0
5000
10000
15000
Seqs/Sample

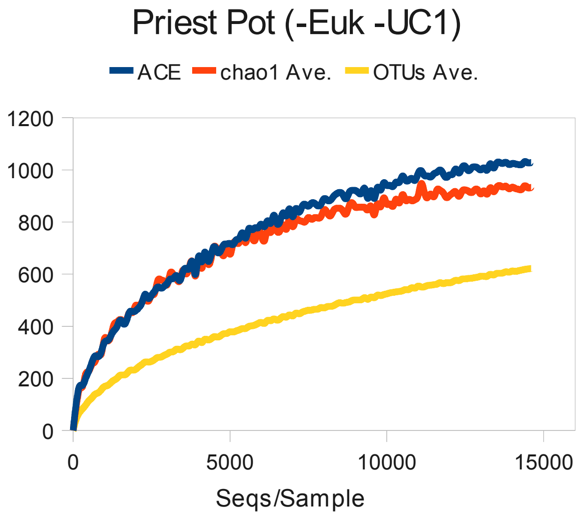
Priest Pot (-Euk -UC1)
ACE
chao1 Ave.
OTUs Ave.
1200
1000
800
600
400
200
0
0
5000
10000
15000
Seqs/Sample

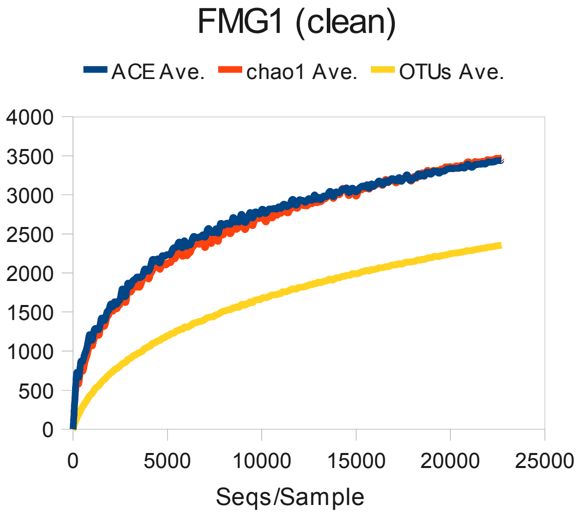
FMG1 (clean)
ACE Ave.
chao1 Ave.
OTUs Ave.
4000
3500
3000
2500
2000
1500
1000
500
0
0
5000
10000
15000
20000
25000
Seqs/Sample

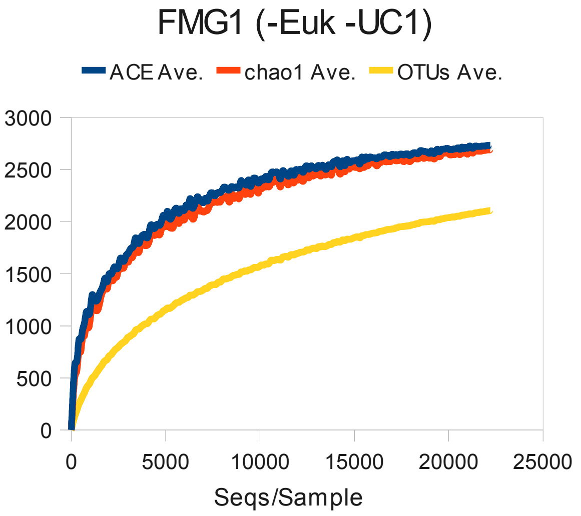
FMG1 (-Euk -UC1)
ACE Ave.
chao1 Ave.
OTUs Ave.
3000
2500
2000
1500
1000
500
0
0
5000
10000
15000
20000
25000
Seqs/Sample

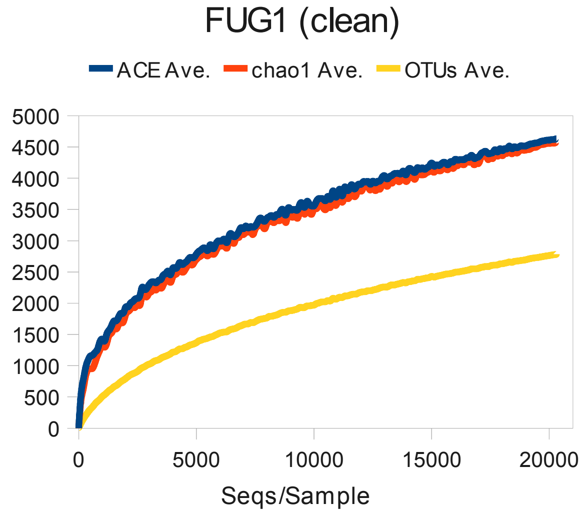
FUG1 (clean)
ACE Ave.
chao1 Ave.
OTUs Ave.
5000
4500
4000
3500
3000
2500
2000
1500
1000
500
0
0
5000
10000
15000
20000
Seqs/Sample

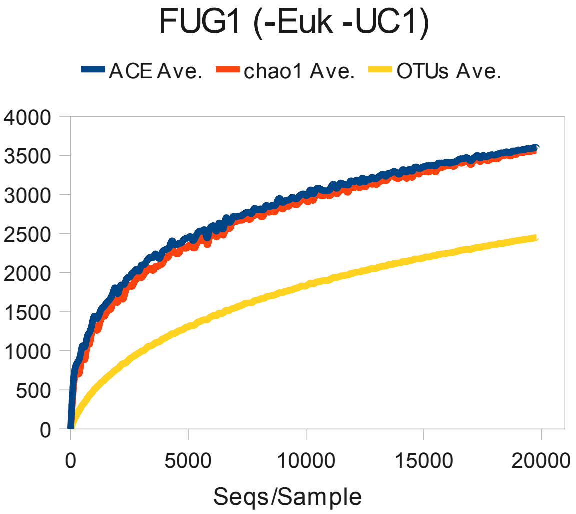
FUG1 (-Euk -UC1)
ACE Ave.
chao1 Ave.
OTUs Ave.
4000
3500
3000
2500
2000
1500
1000
500
0
0
5000
10000
15000
20000
Seqs/Sample

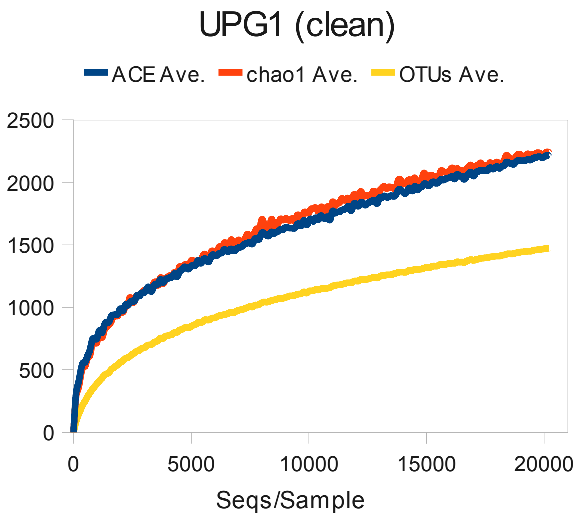
UPG1 (clean)
ACE Ave.
chao1 Ave.
OTUs Ave.
0
500
1000
1500
2000
2500
0
5000
10000
15000
20000
Seqs/Sample

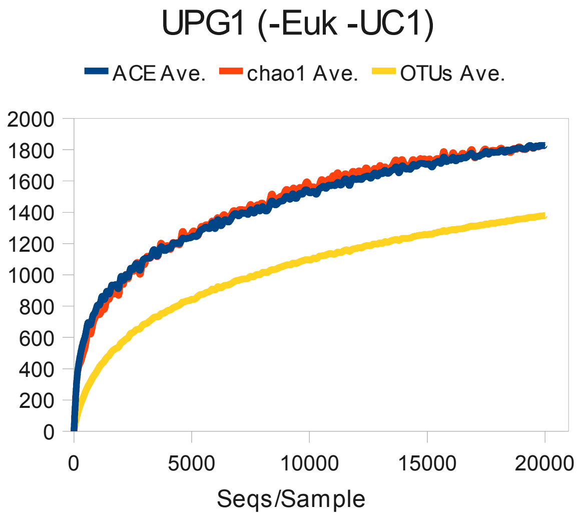
UPG1 (-Euk -UC1)
ACE Ave.
chao1 Ave.
OTUs Ave.
0
200
400
600
800
1000
1200
1400
1600
1800
2000
0
5000
10000
15000
20000
Seqs/Sample

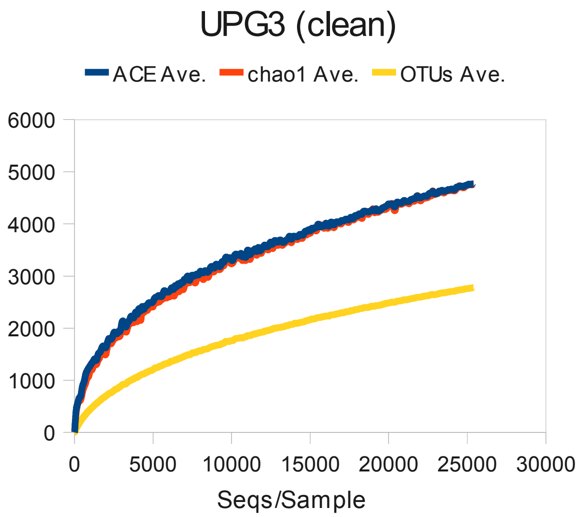
UPG3 (clean)
ACE Ave.
chao1 Ave.
OTUs Ave.
0
1000
2000
3000
4000
5000
6000
0
5000
10000
15000
20000
25000
30000
Seqs/Sample

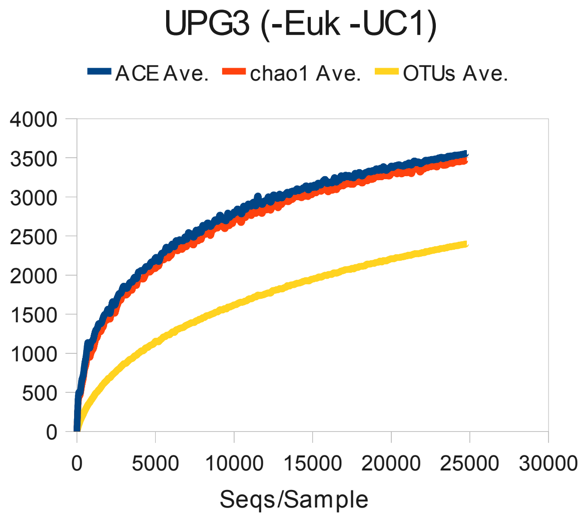
UPG3 (-Euk -UC1)
ACE Ave.
chao1 Ave.
OTUs Ave.
0
500
1000
1500
2000
2500
3000
3500
4000
0
5000
10000
15000
20000
25000
30000
Seqs/Sample

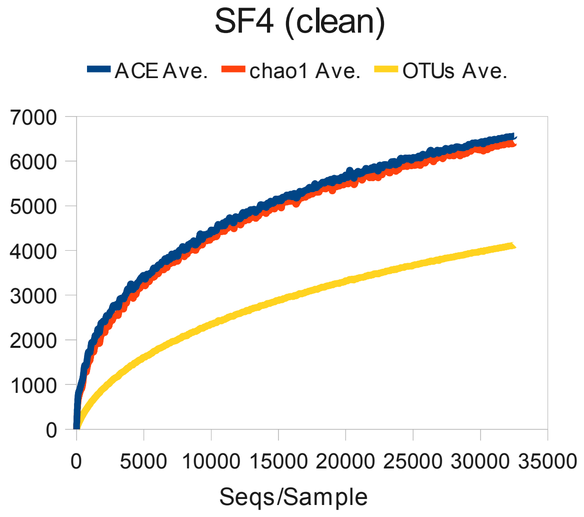
SF4 (clean)
ACE Ave.
chao1 Ave.
OTUs Ave.
0
1000
2000
3000
4000
5000
6000
7000
0
5000
10000
15000
20000
25000
30000
35000
Seqs/Sample

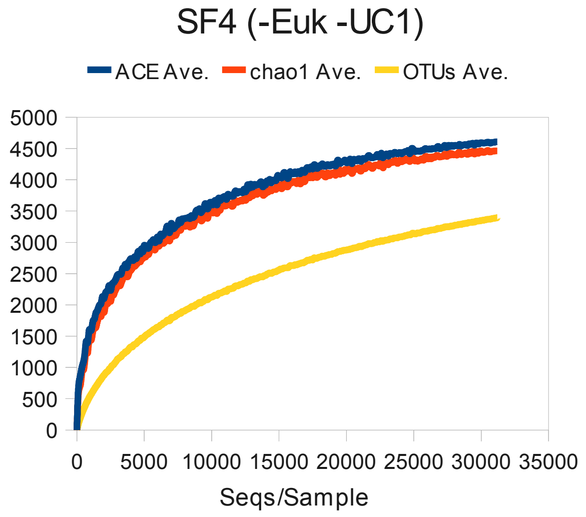
SF4 (-Euk -UC1)
ACE Ave.
chao1 Ave.
OTUs Ave.
0
500
1000
1500
2000
2500
3000
3500
4000
4500
5000
0
5000
10000
15000
20000
25000
30000
35000
Seqs/Sample

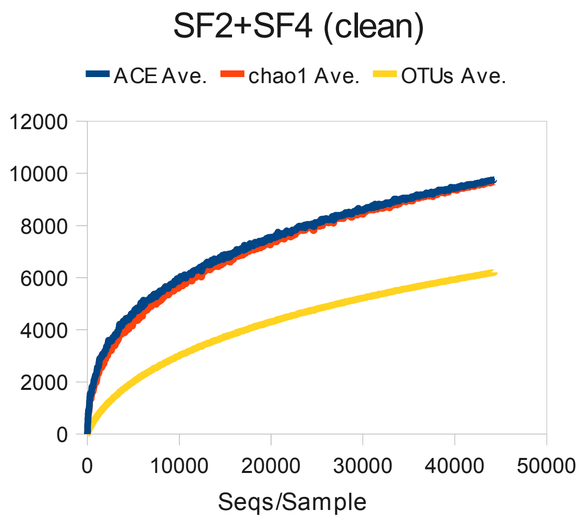
SF2+SF4 (clean)
ACE Ave.
chao1 Ave.
OTUs Ave.
0
2000
4000
6000
8000
10000
12000
0
10000
20000
30000
40000
50000
Seqs/Sample

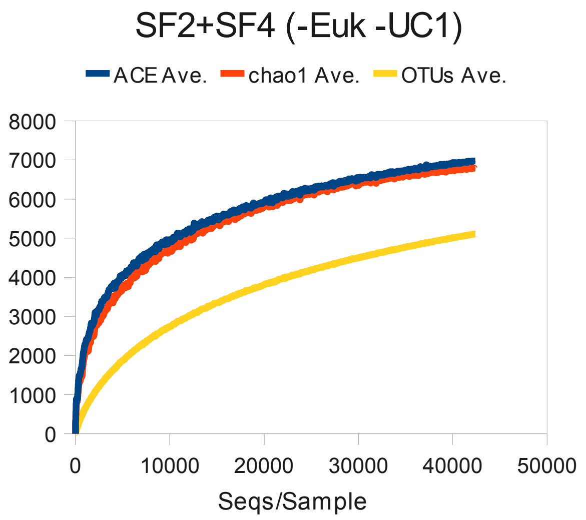
SF2+SF4 (-Euk -UC1)
ACE Ave.
chao1 Ave.
OTUs Ave.
0
1000
2000
3000
4000
5000
6000
7000
8000
0
10000
20000
30000
40000
50000
Seqs/Sample

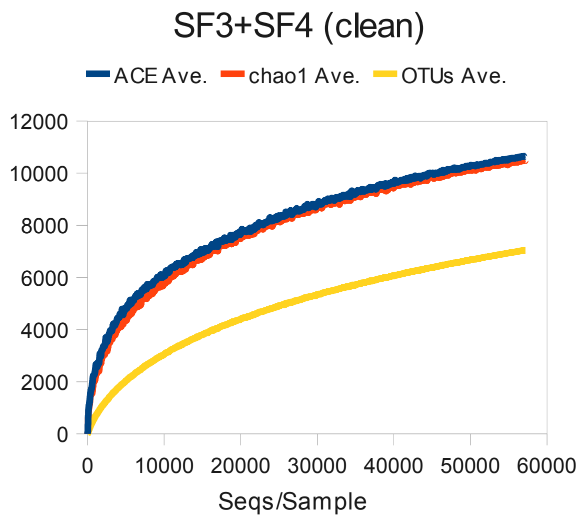
SF3+SF4 (clean)
ACE Ave.
chao1 Ave.
OTUs Ave.
0
2000
4000
6000
8000
10000
12000
0
10000
20000
30000
40000
50000
60000
Seqs/Sample

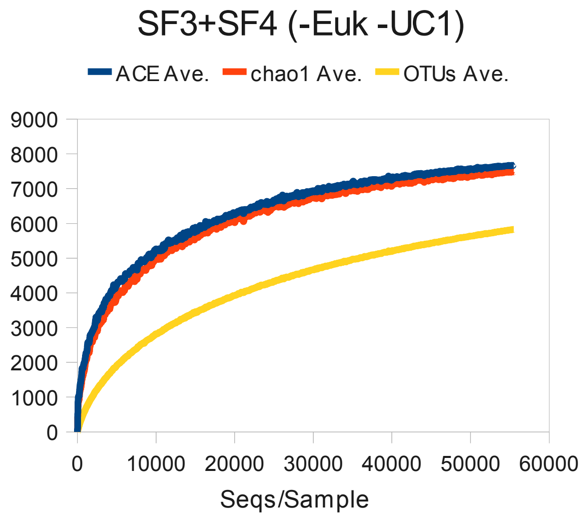
SF3+SF4 (-Euk -UC1)
ACE Ave.
chao1 Ave.
OTUs Ave.
0
1000
2000
3000
4000
5000
6000
7000
8000
9000
0
10000
20000
30000
40000
50000
60000
Seqs/Sample

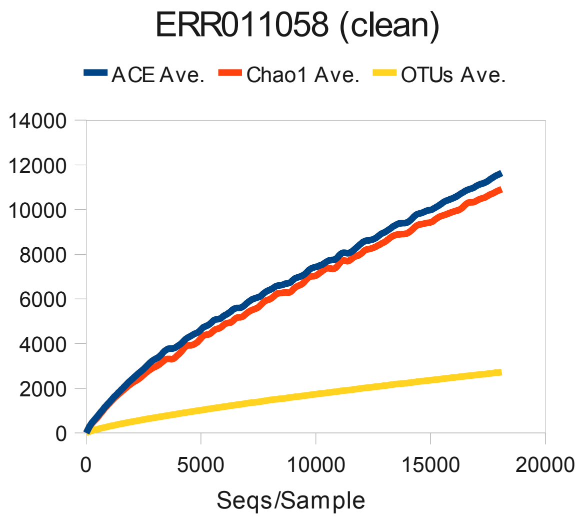
ERR011058 (clean)
ACE Ave.
Chao1 Ave.
OTUs Ave.
0
2000
4000
6000
8000
10000
12000
14000
0
5000
10000
15000
20000
Seqs/Sample

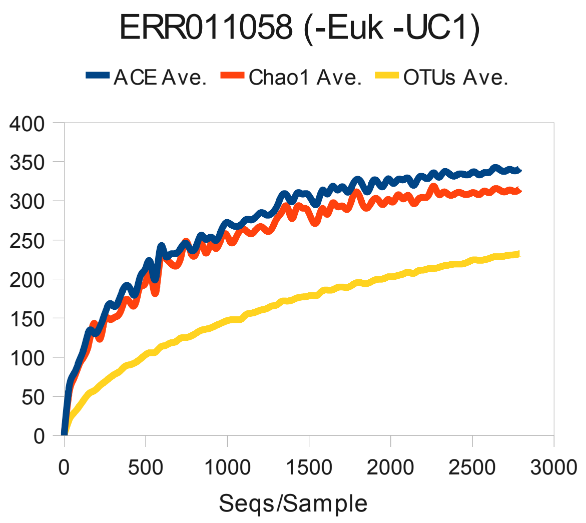
ERR011058 (-Euk -UC1)
ACE Ave.
Chao1 Ave.
OTUs Ave.
0
50
100
150
200
250
300
350
400
0
500
1000
1500
2000
2500
3000
Seqs/Sample

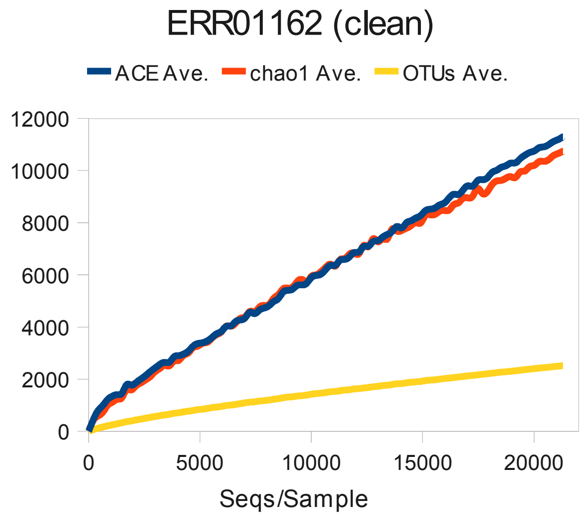
ERR01162 (clean)
ACE Ave.
chao1 Ave.
OTUs Ave.
12000
10000
8000
6000
4000
2000
0
0
5000
10000
15000
20000
Seqs/Sample

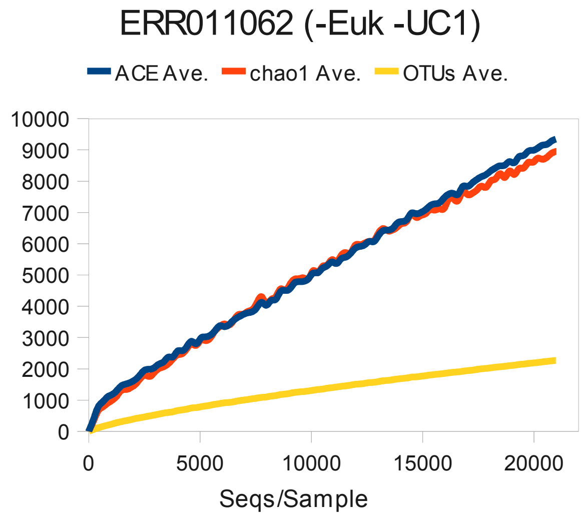
ERR011062 (-Euk -UC1)
ACE Ave.
chao1 Ave.
OTUs Ave.
10000
9000
8000
7000
6000
5000
4000
3000
2000
1000
0
0
5000
10000
15000
20000
Seqs/Sample

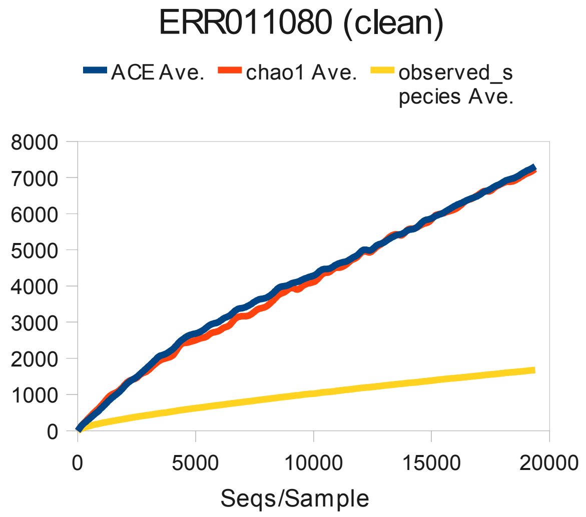
ERR011080 (clean)
ACE Ave.
chao1 Ave.
observed_species Ave.
8000
7000
6000
5000
4000
3000
2000
1000
0
0
5000
10000
15000
20000
Seqs/Sample

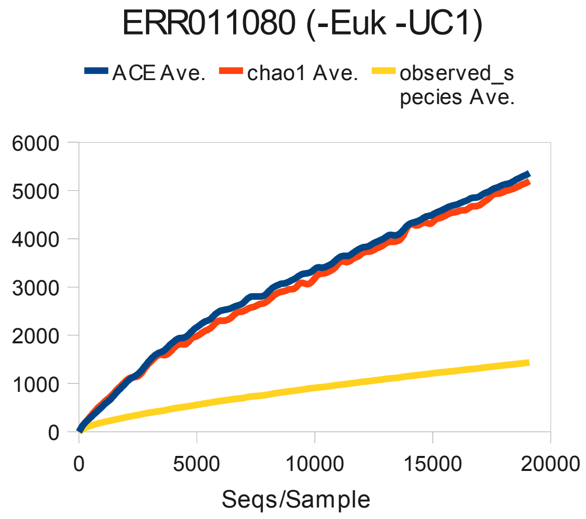
ERR011080 (-Euk -UC1)
ACE Ave.
chao1 Ave.
observed_species Ave.
6000
5000
4000
3000
2000
1000
0
0
5000
10000
15000
20000
Seqs/Sample
