# Supplementary material for: Analysis of Metagenomic Data Containing High Biodiversity Levels
Source: PLoS One. 2013 Mar 7;8(3):e58118. doi: 10.1371/journal.pone.0058118 (PMC3591453; doi:10.1371/journal.pone.0058118)
Supplement: Table S1 — Average values of Chao1 for all datasets. The average and the last ten observed values of Chao1 and their corresponding number of sampled sequences grouped in each case are listed to provide a more accurate picture of its evolution at the end of the rarefaction curve. (DOC) [file pone.0058118.s004.doc]

| **Priest Pot clean** | | **FMG1 clean** | | **FUG1 clean** | | **UPG1 clean** | | **UPG3 clean** | | **SF4 clean** | | **SF2+SF4 clean** | | **SF3+SF4 clean** | | **ERR011058 clean** | | **ERR011062 clean** | | **ERR011080 clean** | |
| --- | --- | --- | --- | --- | --- | --- | --- | --- | --- | --- | --- | --- | --- | --- | --- | --- | --- | --- | --- | --- | --- |
| Chao1 | Reads | Chao1 | Reads | Chao1 | Reads | Chao1 | Reads | Chao1 | Reads | Chao1 | Reads | Chao1 | Reads | Chao1 | Reads | Chao1 | Reads | Chao1 | Reads | Chao1 | Reads |
| 1395 | 14200 | 3404 | 21800 | 4491 | 19400 | 2228 | 19300 | 4734 | 24500 | 6382 | 31600 | 9622 | 43400 | 10451 | 56300 | 10167 | 16471 | 9949 | 19383 | 6753 | 17654 |
| 1402 | 14300 | 3437 | 21900 | 4511 | 19500 | 2208 | 19400 | 4710 | 24600 | 6361 | 31700 | 9652 | 43500 | 10449 | 56400 | 10384 | 16652 | 9984 | 19596 | 6777 | 17848 |
| 1407 | 14400 | 3448 | 22000 | 4533 | 19600 | 2223 | 19500 | 4696 | 24700 | 6376 | 31800 | 9631 | 43600 | 10451 | 56500 | 10263 | 16833 | 10152 | 19809 | 6853 | 18042 |
| 1404 | 14500 | 3435 | 22100 | 4538 | 19700 | 2223 | 19600 | 4693 | 24800 | 6388 | 31900 | 9662 | 43700 | 10472 | 56600 | 10407 | 17014 | 10206 | 20022 | 6906 | 18236 |
| 1407 | 14600 | 3449 | 22200 | 4546 | 19800 | 2236 | 19700 | 4732 | 24900 | 6396 | 32000 | 9629 | 43800 | 10472 | 56700 | 10506 | 17195 | 10349 | 20235 | 6863 | 18430 |
| 1402 | 14700 | 3444 | 22300 | 4558 | 19900 | 2233 | 19800 | 4736 | 25000 | 6380 | 32100 | 9642 | 43900 | 10469 | 56800 | 10501 | 17376 | 10351 | 20448 | 6957 | 18624 |
| 1412 | 14800 | 3463 | 22400 | 4555 | 20000 | 2222 | 19900 | 4755 | 25100 | 6400 | 32200 | 9672 | 44000 | 10480 | 56900 | 10691 | 17557 | 10421 | 20661 | 7023 | 18818 |
| 1406 | 14900 | 3468 | 22500 | 4562 | 20100 | 2227 | 20000 | 4751 | 25200 | 6384 | 32300 | 9660 | 44100 | 10475 | 57000 | 10716 | 17738 | 10577 | 20874 | 7118 | 19012 |
| 1410 | 15000 | 3465 | 22600 | 4561 | 20200 | 2242 | 20100 | 4747 | 25300 | 6393 | 32400 | 9665 | 44200 | 10478 | 57100 | 10842 | 17919 | 10657 | 21087 | 7148 | 19205 |
| 1411 | 15100 | 3472 | 22700 | 4580 | 20300 | 2240 | 20200 | 4763 | 25400 | 6405 | 32500 | 9686 | 44300 | 10486 | 57200 | 10910 | 18100 | 10744 | 21300 | 7233 | 19400 |
|  |  |  |  |  |  |  |  |  |  |  |  |  |  |  |  |  |  |  |  |  |  |
| **Priest Pot -euk -uc1** | | **FMG1 -euk-uc1** | | **FUG1 -euk -uc1** | | **UPG1 -euk -uc1** | | **UPG3 -euk -uc1** | | **SF4 -euk -uc1** | | **SF2+SF4 -euk -uc1** | | **SF3+SF4 -euk -uc1** | | **ERR011058 -euk -uc1** | | **ERR011062 -euk -uc1** | | **ERR011080 -euk -uc1** | |
| Chao1 | Reads | Chao1 | Reads | Chao1 | Reads | Chao1 | Reads | Chao1 | Reads | Chao1 | Reads | Chao1 | Reads | Chao1 | Reads | Chao1 | Reads | Chao1 | Reads | Chao1 | Reads |
| 934 | 13700 | 2659 | 21300 | 3509 | 18900 | 1802 | 19100 | 3426 | 23900 | 4446 | 30300 | 6755 | 41500 | 7473 | 54600 | 8265 | 16198 | 8210 | 19110 | 4804 | 17381 |
| 937 | 13800 | 2682 | 21400 | 3531 | 19000 | 1803 | 19200 | 3454 | 24000 | 4458 | 30400 | 6793 | 41600 | 7470 | 54700 | 8246 | 16376 | 8401 | 19320 | 4967 | 17572 |
| 927 | 13900 | 2678 | 21500 | 3536 | 19100 | 1820 | 19300 | 3445 | 24100 | 4469 | 30500 | 6785 | 41700 | 7482 | 54800 | 8381 | 16554 | 8419 | 19530 | 4914 | 17763 |
| 933 | 14000 | 2676 | 21600 | 3538 | 19200 | 1823 | 19400 | 3452 | 24200 | 4448 | 30600 | 6779 | 41800 | 7484 | 54900 | 8448 | 16732 | 8597 | 19740 | 4954 | 17954 |
| 928 | 14100 | 2675 | 21700 | 3546 | 19300 | 1808 | 19500 | 3447 | 24300 | 4445 | 30700 | 6790 | 41900 | 7470 | 55000 | 8535 | 16910 | 8602 | 19950 | 5010 | 18145 |
| 925 | 14200 | 2682 | 21800 | 3561 | 19400 | 1819 | 19600 | 3452 | 24400 | 4455 | 30800 | 6771 | 42000 | 7471 | 55100 | 8498 | 17088 | 8733 | 20160 | 5011 | 18336 |
| 925 | 14300 | 2682 | 21900 | 3546 | 19500 | 1817 | 19700 | 3456 | 24500 | 4456 | 30900 | 6795 | 42100 | 7482 | 55200 | 8610 | 17266 | 8699 | 20370 | 5053 | 18527 |
| 939 | 14400 | 2688 | 22000 | 3559 | 19600 | 1823 | 19800 | 3456 | 24600 | 4459 | 31000 | 6795 | 42200 | 7479 | 55300 | 8720 | 17444 | 8766 | 20580 | 5111 | 18718 |
| 930 | 14500 | 2687 | 22100 | 3565 | 19700 | 1827 | 19900 | 3469 | 24700 | 4463 | 31100 | 6791 | 42300 | 7481 | 55400 | 8755 | 17622 | 8893 | 20790 | 5147 | 18909 |
| 933 | 14600 | 2690 | 22200 | 3568 | 19800 | 1825 | 20000 | 3467 | 24800 | 4461 | 31200 | 6790 | 42400 | 7482 | 55500 | 8838 | 17800 | 8957 | 21000 | 5191 | 19100 |
