# Supplementary material for: Analysis of Metagenomic Data Containing High Biodiversity Levels
Source: PLoS One. 2013 Mar 7;8(3):e58118. doi: 10.1371/journal.pone.0058118 (PMC3591453; doi:10.1371/journal.pone.0058118)
Supplement: Table S2 — Frequencies of taxonomic grouped sequences in each dataset. For each dataset the following data are provided: total number of sequences remaining after quality filtering and removal of chimeras and sequences that are too short (N), number of sequences identified as being of eukaryotic origin (Eukaryota), number of sequences identified as being of bacterial origin (Bacteria), number of sequences that could not truly be assigned (Unclassified) and proportion of unclassified sequences relative to the total number of sequences (U/N). (DOC) [file pone.0058118.s005.doc]

| **Dataset** | **N** | **Eukaryota** | **Bacteria** | **Unclassified** | **U/N** |
| --- | --- | --- | --- | --- | --- |
| synthetic 16S | 20001 | 5 | 15486 | 4510 | 0.23 |
| SF4 maize soil | 34080 | 44 | 27089 | 6947 | 0.20 |
| SF2+SF4 maize soil | 46142 | 24 | 36978 | 9140 | 0.20 |
| SF3+SF4 maize soil | 59656 | 70 | 49353 | 10233 | 0.17 |
| Priest Pot lake | 15553 | 156 | 12591 | 2806 | 0.18 |
| FMG1 grassland | 23292 | 5 | 20855 | 2432 | 0.10 |
| FUG1 grassland | 21061 | 24 | 18074 | 2963 | 0.14 |
| UPG1 grassland | 20888 | 21 | 18524 | 2343 | 0.11 |
| UPG3 grassland | 26057 | 30 | 22948 | 3079 | 0.12 |
| ERR011058 | 18152 | 0 | 14643 | 3509 | 0.19 |
| ERR011062 | 21378 | 0 | 18723 | 2655 | 0.12 |
| ERR011080 | 19412 | 0 | 14741 | 4671 | 0.24 |
